# Epidemiology of facial fractures: incidence, prevalence and years lived with disability estimates from the Global Burden of Disease 2017 study

**DOI:** 10.1136/injuryprev-2019-043297

**Published:** 2020-01-08

**Authors:** Ratilal Lalloo, Lydia R Lucchesi, Catherine Bisignano, Chris D Castle, Zachary V Dingels, Jack T Fox, Erin B Hamilton, Zichen Liu, Nicholas L S Roberts, Dillon O Sylte, Fares Alahdab, Vahid Alipour, Ubai Alsharif, Jalal Arabloo, Mojtaba Bagherzadeh, Maciej Banach, Ali Bijani, Christopher Stephen Crowe, Ahmad Daryani, Huyen Phuc Do, Linh Phuong Doan, Florian Fischer, Gebreamlak Gebremedhn Gebremeskel, Juanita A Haagsma, Arvin Haj-Mirzaian, Arya Haj-Mirzaian, Samer Hamidi, Chi Linh Hoang, Seyed Sina Naghibi Irvani, Amir Kasaeian, Yousef Saleh Khader, Rovshan Khalilov, Abdullah T Khoja, Aliasghar A Kiadaliri, Marek Majdan, Navid Manaf, Ali Manafi, Benjamin Ballard Massenburg, Abdollah Mohammadian-Hafshejani, Shane Douglas Morrison, Trang Huyen Nguyen, Son Hoang Nguyen, Cuong Tat Nguyen, Tinuke O Olagunju, Nikita Otstavnov, Suzanne Polinder, Navid Rabiee, Mohammad Rabiee, Kiana Ramezanzadeh, Kavitha Ranganathan, Aziz Rezapour, Saeed Safari, Abdallah M Samy, Lidia Sanchez Riera, Masood Ali Shaikh, Bach Xuan Tran, Parviz Vahedi, Amir Vahedian-Azimi, Zhi-Jiang Zhang, David M Pigott, Simon I Hay, Ali H Mokdad, Spencer L James

**Affiliations:** 1 School of Dentistry, The University of Queensland, Brisbane, Queensland, Australia; 2 Institute for Health Metrics and Evaluation, University of Washington, Seattle, Washington, USA; 3 Evidence Based Practice Center, Mayo Clinic Foundation for Medical Education and Research, Rochester, Minnesota, USA; 4 Health Management and Economics Research Center, Tehran, Iran; 5 Health Economics Department, Iran University of Medical Sciences, Tehran, Iran; 6 Department of Oral and Maxillofacial Surgery, University Hospital Knappschaftskrankenhaus Bochum, Bochum, Germany; 7 Health Management and Economics Research Center, Iran University of Medical Sciences, Tehran, Iran; 8 Chemistry Department, Sharif University of Technology, Tehran, Iran; 9 Department of Hypertension, Medical University of Lodz, Lodz, Poland; 10 Polish Mothers’ Memorial Hospital Research Institute, Lodz, Poland; 11 Social Determinants of Health Research Center, Babol University of Medical Sciences, Babol, Iran; 12 Division of Plastic Surgery, University of Washington, Seattle, Washington, USA; 13 Toxoplasmosis Research Center, Mazandaran University of Medical Sciences, Sari, Iran; 14 Center of Excellence in Behavioral Medicine, Nguyen Tat Thanh University, Ho Chi Minh, Vietnam; 15 Center of Excellence in Health Service Management, Nguyen Tat Thanh University, Ho Chi Minh, Vietnam; 16 School of Public Health Medicine, Bielefeld University, Bielefeld, Germany; 17 Nursing Department, College of Health Science, Aksum University, Aksum, Ethiopia; 18 Nursing Department, Mekelle University, Mekelle, Ethiopia; 19 Department of Public Health, Erasmus University Medical Center, Rotterdam, The Netherlands; 20 Department of Pharmacology, Tehran University of Medical Sciences, Tehran, Iran; 21 Obesity Research Center, Shahid Beheshti University of Medical Sciences, Tehran, Iran; 22 Department of Radiology, Johns Hopkins University, Baltimore, Maryland, USA; 23 School of Health and Environmental Studies, Hamdan Bin Mohammed Smart University, Dubai, United Arab Emirates; 24 Research Institute for Endocrine Sciences, Shahid Beheshti University of Medical Sciences, Tehran, Iran; 25 Hematology-Oncology and Stem Cell Transplantation Research Center, Tehran University of Medical Sciences, Tehran, Iran; 26 Hematologic Malignancies Research Center, Tehran University of Medical Sciences, Tehran, Iran; 27 Department of Public Health and Community Medicine, Jordan University of Science and Technology, Ramtha, Jordan; 28 Department of Physiology, Baku State University, Baku, Azerbaijan; 29 Department of Public Health, Imam Muhammad Ibn Saud Islamic University, Riyadh, Saudi Arabia; 30 Department of Health Policy and Management, Johns Hopkins University, Baltimore, Maryland, USA; 31 Clinical Epidemiology Unit, Lund University, Lund, Sweden; 32 Department of Public Health, Trnava University, Trnava, Slovakia; 33 Ophthalmology Department, Iran University of Medical Sciences, Tehran, Iran; 34 Ophthalmology Department, University of Manitoba, Winnipeg, Manitoba, Canada; 35 Plastic Surgery Department, Iran University of Medical Sciences, Tehran, Iran; 36 Department of Epidemiology and Biostatistics, Shahrekord University of Medical Sciences, Shahrekord, Iran; 37 Department of Surgery, University of Washington, Seattle, Washington, USA; 38 Institute for Global Health Innovations, Duy Tan University, Hanoi, Vietnam; 39 Department of Pathology and Molecular Medicine, McMaster University, Hamilton, Ontario, Canada; 40 Laboratory of Public Health Indicators Analysis and Health Digitalization, Moscow Institute of Physics and Technology, Dolgoprudny, Russia; 41 Academic Department, Unium Ltd, Moscow, Russia; 42 Biomedical Engineering Department, Amirkabir University of Technology, Tehran, Iran; 43 Department of Pharmacology, Shahid Beheshti University of Medical Sciences, Tehran, Iran; 44 Department of Surgery, University of Michigan, Ann Arbor, Michigan, USA; 45 Emergency Department, Shahid Beheshti University of Medical Sciences, Tehran, Iran; 46 Department of Entomology, Ain Shams University, Cairo, Egypt; 47 Rheumatology Department, University Hospitals Bristol NHS Foundation Trust, Bristol, UK; 48 Institute of Bone and Joint Research, University of Sydney, Syndey, New South Wales, Australia; 49 Independent Consultant, Karachi, Pakistan; 50 Department of Health Economics, Hanoi Medical University, Hanoi, Vietnam; 51 Assistant Professor of Anatomical Sciences, Maragheh University of Medical Sciences, Maragheh, Iran; 52 Trauma Research Center, Nursing Faculty, Baqiyatallah University of Medical Sciences, Tehran, Iran; 53 Department of Preventive Medicine, Wuhan University, Wuhan, China; 54 Department of Health Metrics Sciences, School of Medicine, University of Washington, Seattle, Washington, USA

**Keywords:** dental injury, burden of disease, descriptive epidemiology

## Abstract

**Background:**

The Global Burden of Disease Study (GBD) has historically produced estimates of causes of injury such as falls but not the resulting types of injuries that occur. The objective of this study was to estimate the global incidence, prevalence and years lived with disability (YLDs) due to facial fractures and to estimate the leading injurious causes of facial fracture.

**Methods:**

We obtained results from GBD 2017. First, the study estimated the incidence from each injury cause (eg, falls), and then the proportion of each cause that would result in facial fracture being the most disabling injury. Incidence, prevalence and YLDs of facial fractures are then calculated across causes.

**Results:**

Globally, in 2017, there were 7 538 663 (95% uncertainty interval 6 116 489 to 9 493 113) new cases, 1 819 732 (1 609 419 to 2 091 618) prevalent cases, and 117 402 (73 266 to 169 689) YLDs due to facial fractures. In terms of age-standardised incidence, prevalence and YLDs, the global rates were 98 (80 to 123) per 100 000, 23 (20 to 27) per 100 000, and 2 (1 to 2) per 100 000, respectively. Facial fractures were most concentrated in Central Europe. Falls were the predominant cause in most regions.

**Conclusions:**

Facial fractures are predominantly caused by falls and occur worldwide. Healthcare systems and public health agencies should investigate methods of all injury prevention. It is important for healthcare systems in every part of the world to ensure access to treatment resources.

## Introduction

Facial fractures can be disabling injuries that may require complex surgical care from reconstructive plastic surgeons or oral-maxillofacial specialists. While sophisticated diagnostics and surgical treatment approaches have been developed and are routinely utilised in high resource healthcare systems, occult facial fractures are frequent, especially with low energy mechanisms, and may be missed on initial trauma surveys across the wide array of possible causes of trauma.^1–4^ Without a high degree of clinical suspicion and proper diagnostic equipment (CT scans with multiplanar reconstruction, panorex films), the diagnosis of facial fracture may be significantly delayed and may only be apparent once swelling has subsided.^5 6^ In certain instances, this can have devastating consequences, for example, an orbital floor fracture with entrapment of extraocular muscle leading to permanent dysfunction of congruent eye movements.^7^ In addition, there may be considerable burden of such injuries in lower resource areas of the world that lack access to timely and effective care, even if surgical intervention is not indicated. In some cases, effective care may involve non-operative management. For instance, a minimally displaced mandibular condyle fracture may be managed with a soft, non-chew diet for several weeks.^8^ In the instance of a mandible fracture, meticulous oral hygiene is imperative to prevent odontogenic infections.^9^ Regions in which dental hygiene is poor and routine dental care is sparse may be predisposed to poor outcomes with conservative management strategies such as this. Thus, it is important to measure and understand how these injuries occur and where they are most concentrated geographically. Such efforts could help lead to improved resource allocation and better health system planning to ensure that people suffering from such injuries have access to the treatment resources that can mitigate the disability of such conditions and could also help emphasise the importance of injury prevention strategies. Consequently, there is likely considerable value in measuring the burden of these conditions.

To date, there has not been a systematic assessment of the global burden of facial fractures that produced estimates for all countries and across all age and sex groups. Existing literature has focused on anatomically based subsets of fracture patterns,^10^ aetiological factors of known facial fractures,^10–12^ a specific age group of interest,^13^ and assessments in limited, specific geographies such as the USA.^4 14^ Some studies, for example, have estimated the proportions of different injurious aetiologies or have examined risk factors such as age and sex for sustaining facial fractures,^12 15^ but do not attempt to estimate or model these trends in areas that lack data. Given the lack of comprehensive assessments of these injuries, it is of interest to estimate the burden of facial fractures due to all causes of injury ranging from interpersonal violence to falls to road injuries.

The Global Burden of Disease Study (GBD) is the most comprehensive effort to date to measure the burden and trends of injury and disease worldwide.^16–21^ GBD produces annual estimates of all-cause mortality, causes of death, non-fatal health outcomes (ie, incidence, prevalence and years lived with disability (YLDs)), and risk factors. For non-fatal health outcomes such as facial fractures, GBD quantifies health loss by incorporating disability weights and prevalence. This is an important advent for measuring the burden of facial fractures given that these injuries may affect quality of life differently than other injuries and diseases, especially with regard to the social importance of facial structure and function.^22^ The GBD framework also measures the burden of each condition across all countries, ages, sexes and for a range of years. Such analysis is also important for facial fractures, since the mechanisms of injury that lead to a fracture may be concentrated in certain locations or age groups. More detailed estimation of the burden of facial fractures would not only strengthen the ability of healthcare systems to adequately plan for and care for this population, but, from a policy standpoint, would also contribute to the body of evidence that could lead to injury prevention programme targeted at the causes of injuries that most commonly lead to facial fractures.

To date, estimates for the facial fracture burden in the GBD framework have not been available as reported results. Instead, the distribution of sequelae was incorporated as part of the analytical process that computed disability, but results were ultimately only provided by the cause of injury, such as falls, and not the type, or ‘nature’ of injury, in this case facial fracture. Here, we describe an approach of estimating sequela-specific non-fatal burden estimates across all causes of injury and then we report the incidence, prevalence and YLDs for facial fractures, as well as the distribution of injurious causes that lead to facial fractures. This study represents an important step forward in terms of increasing the level of detail provided in GBD estimates.

## Methods

This study’s approach to measuring facial fractures was developed within the existing GBD framework.^16–21^ A summary of key GBD methods is provided in online supplementary appendix 1, and more detailed methods including detailed injury modelling methods are described in the GBD 2017 capstone publications.^16–21^ Our measurement of the burden of facial fractures included two custom analytic components as follows.

10.1136/injuryprev-2019-043297.supp1 Supplementary data

First, GBD categorises facial fractures as being a nature of injury as opposed to a *cause* of injury. The specific case definition for facial fractures in GBD includes fractures to nasal bones, orbits, mandible, maxilla and other facial bones, as coded in ICD9 codes 802 and ICD10 codes S02.2, S02.3, S02.4, S02.5, S02.6, S02.7. The incidence, prevalence and YLDs of these facial fractures have previously been included under each external cause estimate (eg, falls, road injuries, interpersonal violence).

Second, facial fractures are only measured in terms of non-fatal burden and therefore in this study we report incidence, prevalence and YLDs, but not cause-specific mortality rates or years of life lost.

Facial fracture estimation was otherwise conducted as follows. First, the incidence rates of 30 different causes of injury are modelled using DisMod MR 2.1, a meta-regression tool that is used extensively in GBD.^17^ These cause models use various data types including surveillance studies, literature studies, hospital discharge records and emergency department records. Each cause model also use cause-specific mortality to predict the incidence of the external cause-of-injury models (eg, falls), which can cause death, though facial fractures are not themselves considered to be a cause of death.

In the next step, we measure the proportion of each cause that lead to a facial fracture being the most disabling nature of injury. For instance, if an individual falls and sustains an abrasion and also sustains a facial fracture, the facial fracture is used to determine the disability suffered by the individual. For this process, we utilised dual-coded clinical data sources where both the cause and nature of injury are coded using ICD9 or ICD10 coding systems. A full list of sources used in this process is provided in table 1. These proportions are then modelled using a Dirichlet regression technique such that the proportions of nature of injury sum to one across all natures for a given cause, such that every injury requiring medical care has some nature of injury assigned based on the dual-coded clinical data sources. The output from this step is incidence for each cause-nature combination; for instance, the incidence of falls that result in facial fracture.

**Table 1:** Sources of clinical records used for calculating cause-nature proportions for facial fractures

Dual-coded data	Source	Description
Argentina Public Hospital Injury Discharges 2007–2011	Directorate of Health Statistics and Information, Ministry of Health (Argentina)	Public hospital records aggregated to the country level
China Injury Comprehensive Surveillance Study 2009–2011	Chinese Center for Disease Control and Prevention (CCDC)	Inpatient data collected as part of an injury surveillance study in several subnational sites in China: Chongqing, Dalian, Ningbo, Songjiang, Wuzhong, Zhanjiang and Zhuhai
China National Injury Surveillance System 2006–2014	CCDC, Ministry of Health (China)	Nationally representative surveillance system of outpatients with injuries
United Kingdom—England Hospital Episode Statistics 2002–2015	National Health Service (NHS) England	Records of inpatient, outpatient and emergency attendances at NHS hospitals in England
Netherlands National Medical Registry (LMR) 1998–2012	Dutch Hospital Data	Cases of inpatient care in Dutch hospitals
Netherlands Injury Surveillance System 1998–2012	Consumer Safety Institute (Netherlands)	Emergency department data from a representative sample of private hospitals in the Netherlands
Argentina Injury Surveillance System Tabulations 2008	National Institute of Epidemiology, National Administration of Laboratories and Health Institutes, Global Burden of Disease 2010 Injury Expert Group	Inpatient administrative records
United States National Hospital Discharge Survey 1990–2006	National Center for Health Statistics, Centers for Disease Control and Prevention	Sample of inpatient records selected from a national sample of non-Federal, short-stay hospitals
Bulgaria Hospital Discharge Injury Tabulations 2004	Global Burden of Disease 2010 Injury Expert Group	Inpatient administrative records
Czech Republic Hospital Discharge Injury Tabulations 2004	Global Burden of Disease 2010 Injury Expert Group	Inpatient administrative records
Denmark Hospital Discharge Injury Tabulations 2005	Global Burden of Disease 2010 Injury Expert Group	Inpatient administrative records
Estonia Hospital Discharge Injury Tabulations 2003	Global Burden of Disease 2010 Injury Expert Group	Inpatient administrative records
Hungary Hospital Discharge Injury Tabulations 2004	Global Burden of Disease 2010 Injury Expert Group	Inpatient administrative records
Iceland Hospital Discharge Injury Tabulations 2005	Global Burden of Disease 2010 Injury Expert Group	Inpatient administrative records
Italy Hospital Discharge Injury Tabulations 2003	Global Burden of Disease 2010 Injury Expert Group	Inpatient administrative records
Latvia Hospital Discharge Injury Tabulations 2004	Global Burden of Disease 2010 Injury Expert Group	Inpatient administrative records
Malta Hospital Discharge Injury Tabulations 2005	Global Burden of Disease 2010 Injury Expert Group	Inpatient administrative records
Netherlands Hospital Discharge Injury Tabulations 2004–2005	Global Burden of Disease 2010 Injury Expert Group	Inpatient administrative records
Norway Hospital Discharge Injury Tabulations 2004	Global Burden of Disease 2010 Injury Expert Group	Inpatient administrative records
Portugal Hospital Discharge Injury Tabulations 2004	Global Burden of Disease 2010 Injury Expert Group	Inpatient administrative records
Slovenia Hospital Discharge Injury Tabulations 2004	Global Burden of Disease 2010 Injury Expert Group	Inpatient administrative records
Sweden Hospital Discharge Injury Tabulations 2004	Global Burden of Disease 2010 Injury Expert Group	Inpatient administrative records
Macedonia Hospital Discharge Injury Tabulations 2005	Global Burden of Disease 2010 Injury Expert Group	Inpatient administrative records
Spain Hospital Discharge Injury Tabulations 2000–2007	Global Burden of Disease 2010 Injury Expert Group	Inpatient administrative records
Mauritius Hospital Discharge Injury Tabulations 2003–2007	Ministry of Health and Quality of Life (Mauritius), Global Burden of Disease 2010 Injury Expert Group	Inpatient administrative records
Mexico Ministry of Health Hospital Discharge Tabulations 2005	Secretariat of Health (Mexico)	Inpatient administrative records
Brazil Hospital Information System 1997–2014	Rio de Janeiro, Brazil: Ministry of Health (Brazil)	Nationally representative administrative discharge records for inpatients and outpatients
Austria Hospital Inpatient Discharges 2001–2010	Federal Ministry of Health (Austria), Statistics Austria	Inpatient administrative records
Canada Discharge Abstract Database 1994–2009	Canadian Institute for Health Information	Hospital administrative data on inpatient discharges from acute care facilities in all Canadian provinces and territories other than Quebec
Mexico Ministry of Health Hospital Discharges 2003–2011	Secretariat of Health (Mexico)	Discharge database from Mexico’s Automated Hospital Discharge System
New Zealand National Minimum Dataset 2000–2014	Ministry of Health (New Zealand)	Hospital discharge data for inpatients and day patients
Chile Hospital Discharges 2001–2011	Santiago, Chile: Ministry of Health (Chile)	Administrative discharge records for inpatients

We then separately model short-term and long-term prevalence estimates using proportions expected to experience short-term versus long-term disability based on long-term follow-up studies.^23–29^ The cause-nature incidence rates are converted to prevalence using the differential equation solver that is used in DisMod MR 2.1. YLDs are then calculated by multiplying the prevalence estimate by the disability weight for each specific nature of injury. Disability weight measurement is described in more detail elsewhere in the GBD literature.^30^ Prevalence, incidence and YLDs for facial fractures are then summed across all causes of injury in order to estimate the all-injury prevalence, incidence and YLDs for facial fractures.

We also present results of facial fracture burden by quintile groupings of countries based on their 2017 Socio-demographic Index (SDI) value, which is a composite measure of lag-distributed income per capita, educational attainment over the age of 15 years, and fertility rate in women under the age of 25.^17^ Additionally, we measured the most common causes of facial fractures in terms of the original cause of injury that led to the disability.

Analyses were completed using Python V.2.7, Stata V.13.1, or R V.3.3. Statistical code used for GBD estimation will be made available on publication.

This study complies with the Guidelines for Accurate and Transparent Health Estimates Reporting recommendations (online supplementary appendix 2).

10.1136/injuryprev-2019-043297.supp2 Supplementary data

## Results

All results are also available via GBD online results tools and visualisations and are publicly available at ghdx.healthdata.org. These resources provide additional detail by cause of injury, age group, sex, year and location.

### Incidence

**Figure 1:**
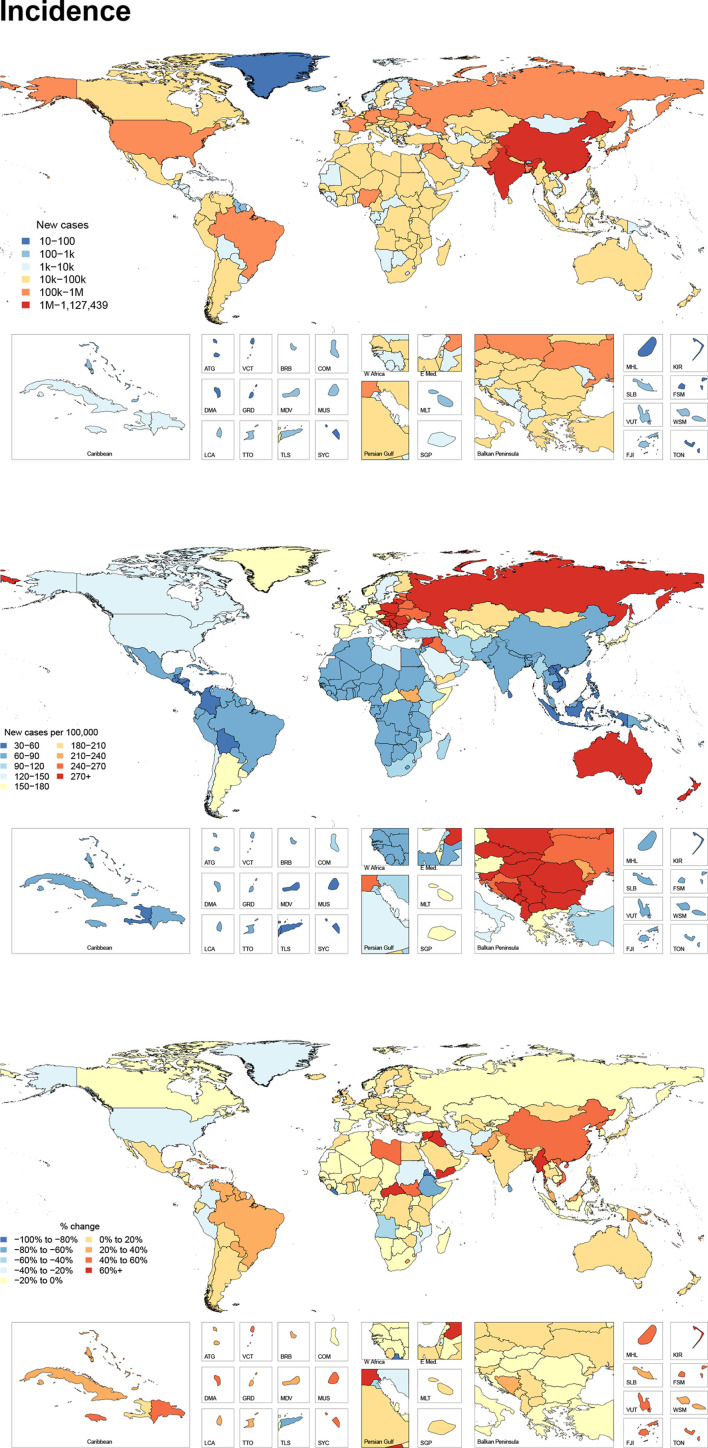
All age new cases, age-standardised incidence and per cent change in age-standardised incidence between 1990 and 2017 of facial fractures per 100 000 by location for both sexes, 2017.

Figure 1 shows the number of new cases for 2017, the age-standardised incidence per 100 000 for 2017, and the per cent change between 1990 and 2017 by country and territory. This figure shows that there are a large number of total cases in populous areas of the world, but that incidence is the highest in the GBD super region of Central Europe, Eastern Europe and Central Asia, with a regional age-standardised incidence of 254 (193 to 335) per 100 000. Within Central Europe, Slovenia had the highest age-standardised incidence rate of 376 (272 to 507) per 100 000, while Poland had the most new cases with 116 518 (84 517 to 161 202) cases in 2017. Select countries in the Middle East, Sub-Saharan Africa and South Asia have also experienced relatively large increases in incidence between 1990 and 2017. Online supplementary appendix table 1 shows the incidence, prevalence and YLDs in terms of all-age counts, age-standardised rates and percentage change from 1990 to 2017 for facial fractures. In 2017, there were an estimated 7 538 663 (95% uncertainty interval (UI) 6 116 489 to 9 493 113) new facial fractures globally. Between 1990 and 2017, the global age-standardised incidence rate did not change significantly. In 2017, it was 98 (80 to 123) per 100 000.

10.1136/injuryprev-2019-043297.supp3 Supplementary data

New cases of facial fractures occur across all SDI quintiles. The high SDI quintile had the highest age-standardised incidence rate of facial fractures at a rate of 158 (122 to 206) per 100 000 while the middle SDI quintile had the lowest with an age-standardised incidence rate of 72 (58 to 89) per 100 000. From 1990 to 2017, age-standardised incidence rates decreased in high and low SDI quintiles, while they increased in low-middle and middle SDI. High-middle SDI had no significant change in incidence.

### Prevalence

**Figure 2:**
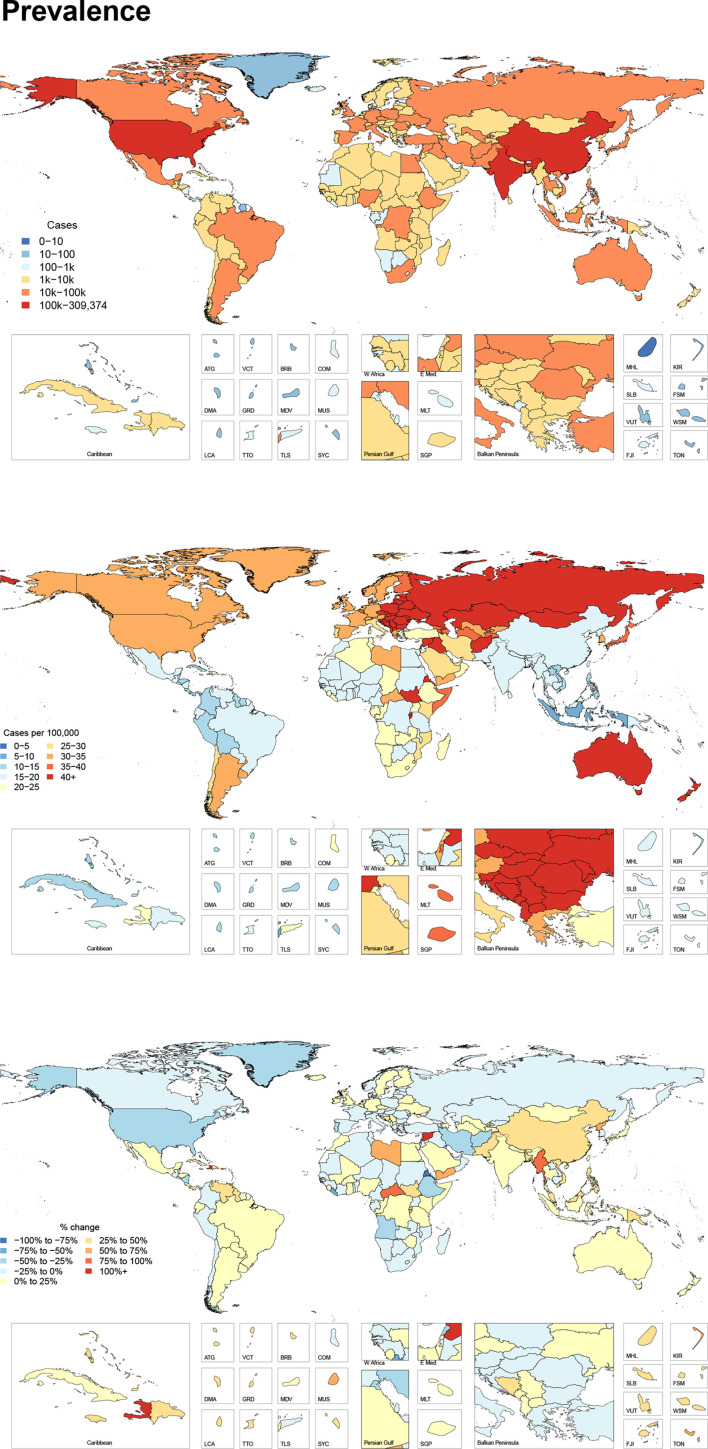
All age cases, age-standardised prevalence and per cent change in age-standardised prevalence between 1990 and 2017 of facial fractures per 100 000 by location for both sexes, 2017.

Figure 2 shows the number of prevalent cases for 2017, the age-standardised prevalence per 100 000 for 2017, and the per cent change between 1990 and 2017 by country. In terms of age-standardised prevalence, the global age-standardised prevalence of facial fractures was 23 (20 to 27) per 100 000 in 2017. This equated to 1 819 732 (1 609 419 to 2 091 618) individuals globally living with any disability from a facial fracture. From 1990 to 2017, there was a significant decrease in the age-standardised prevalence of facial fractures by 2.8% (1.4%–4.1%).

Prevalent cases of facial fractures were distributed across all SDI quintiles in a pattern similar to incident cases. The highest age-standardised prevalence was also in the high SDI quintile with 35 (30 to 41) cases per 100 000, and the lowest was in the middle SDI quintile with 17 (15 to 19) cases per 100 000.

The geographic distribution of prevalent cases was also similar to that of incident cases. In 2017, the age-standardised prevalence of facial fractures was highest in Central Europe with 68 cases (57 to 82) per 100 000, representing 92 387 (80 541 to 108 397) prevalent cases. Within Central Europe, Slovenia and Czech Republic had the highest age-standardised prevalence with identical prevalences of 81 (69 to 99) cases per 100 000, while Poland had the highest total number of prevalent cases with 31 345 (27 039 to 36 935) total cases in 2017.

### Age patterns of incidence and prevalence

**Figure 3:**
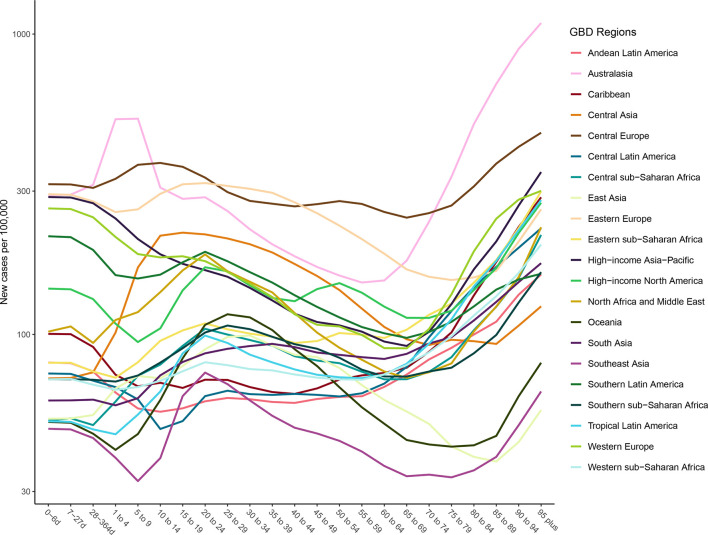
Age-specific incidence of facial fractures per 100 000 by region and age for both sexes, 2017.

**Figure 4:**
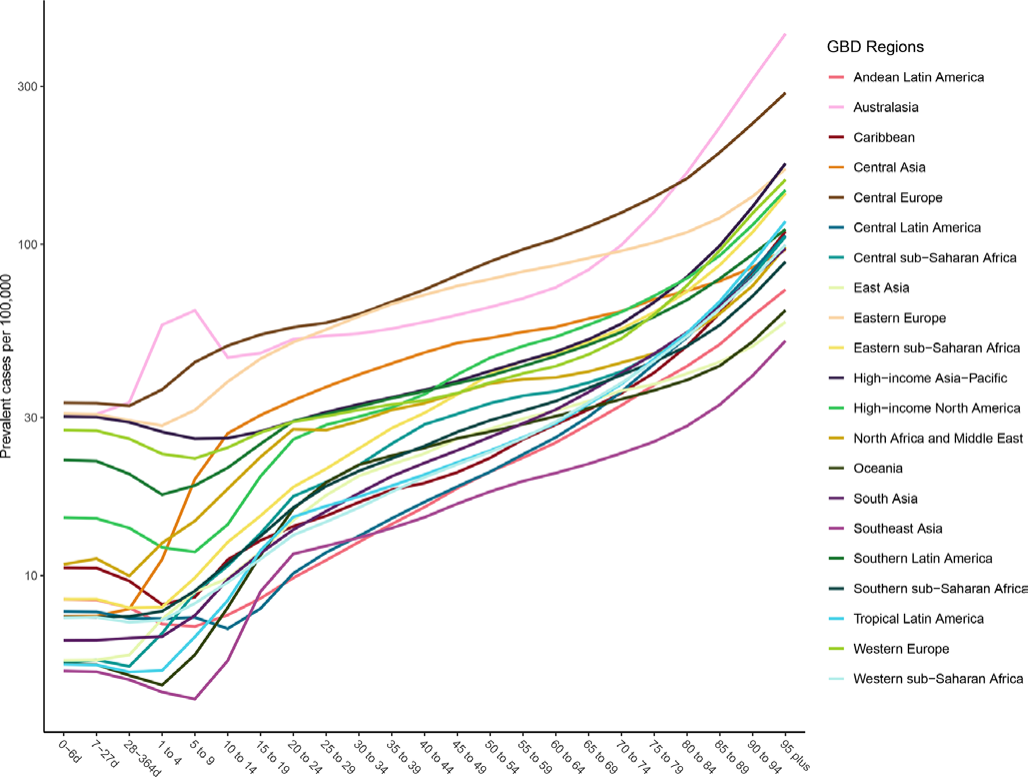
Age-specific prevalence of facial fractures per 100 000 by region and age for both sexes, 2017.

Figures 3 and 4 show the age-specific incidence and prevalence of facial fractures by GBD region, respectively. Incident cases rise in most regions from ages 5 to 20 and rise again in the 70+ age groups. A few regions, like Western Europe and Central Latin America, have distinct age-specific patterns. Figure 3 shows that prevalence of facial fractures increases with age and is the highest in the Australasia, Eastern Europe and Central Europe.

### Years lived with disability

Globally, facial fractures caused 117 402 (73 266 to 169 689) YLDs in 2017. The average disability weight across all ages, sexes and locations was approximately 6.5%, meaning that on average each person with a prevalent facial fracture lost 6.5% of their normal health status. The age-standardised YLD rates globally and by country and territory were all relatively low, with fewer than 10 YLDs per 100 000 in every location in 2017. The age-standardised YLD rates decreased significantly in the high and high-middle SDI quintiles and increased significantly in the middle and low-middle SDI quintiles. The geographic distributions of YLDs were similar to those for incidence and prevalence, as described above.

### Cause of facial fractures

**Figure 5:**
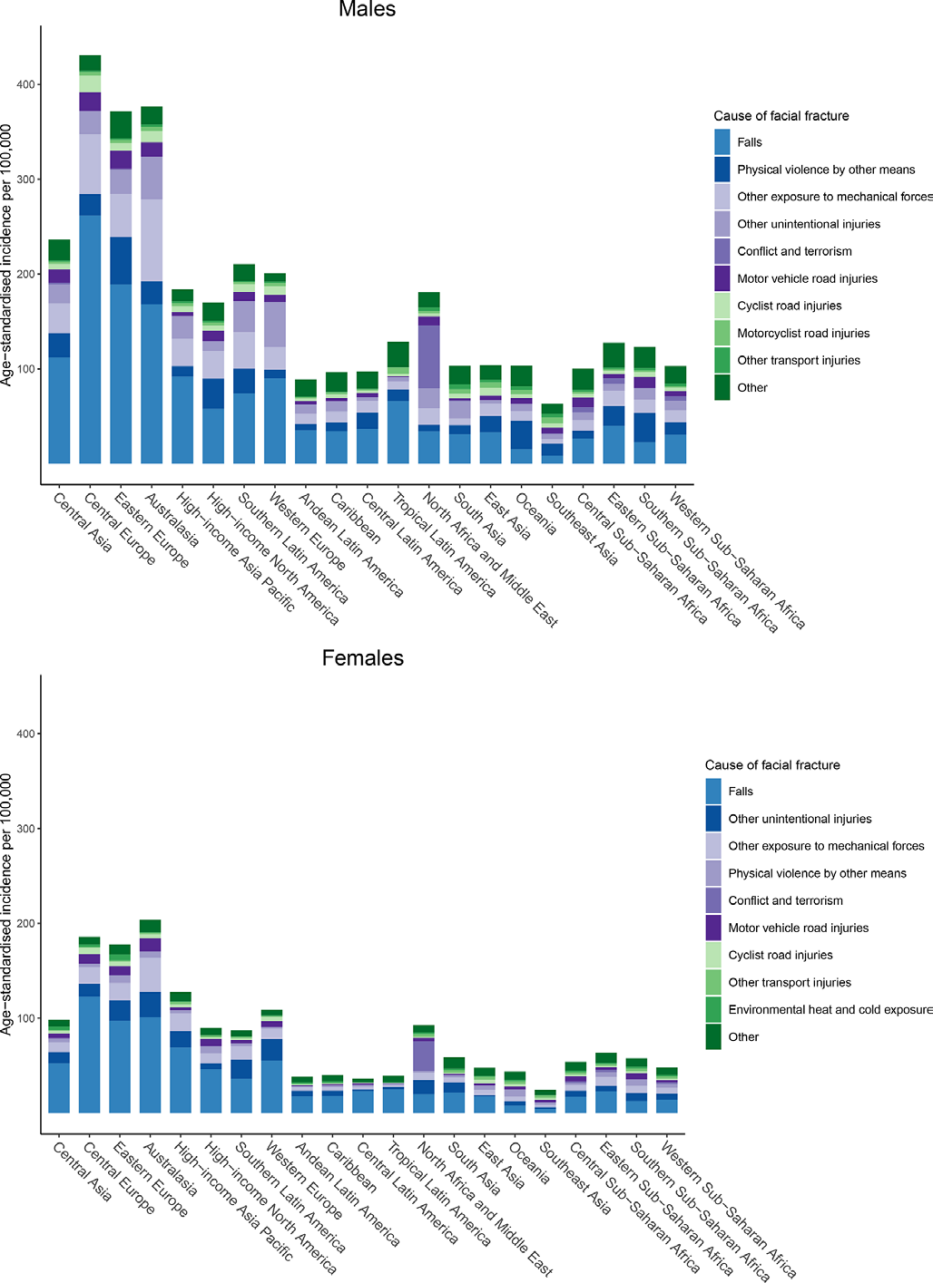
External cause composition of age-standardised incidence of facial fracture by Global Burden of Disease region.

The external causes of the injuries that led to YLDs from facial fracture varied by geographical region and sex, as shown in figure 5. We found that falls were generally the leading driver of age-standardised incidence rates of facial fractures for both sexes, though certain regions such as Oceania and southern sub-Saharan Africa had higher rates from physical violence by other means for males. The proportions due to falls were particularly high in the regions with high facial fracture burden, specifically Central and Eastern Europe. Physical violence by other means, other exposure to mechanical forces, and other unintentional injuries were also important causes of facial fractures in both sexes. In the North Africa and Middle East region, conflict and terrorism was the leading cause of facial fractures in 2017 in both sexes.

## Discussion

This is the first known study to systematically measure the burden of facial fractures from every injurious cause for every country, age group and sex over a study period of several decades. The findings from this study can be organised into three overarching points. First, the burden of facial fractures is distributed across a wide span of geographies and income groups. Whereas some communicable diseases are concentrated in certain regions of the world or some non-communicable diseases become more common after a country experiences an epidemiological transition, injuries, and in this case facial fractures, occur ubiquitously. This is perhaps unsurprising as there are various traumatic mechanisms and risk factors of facial fractures that are unrelated to region or SDI. Nevertheless, this highlights the importance of every country and income group in the world having injury prevention strategies, particularly for causes such as falls,^31–33^ as well as access to medical and surgical care to both diagnose and treat facial fractures that require intervention. Such prevention and care resources are likely more available in higher income areas of the world, and lower resource healthcare systems should ensure that their populations have access to adequate specialist care for managing these injuries. While the burden of facial fractures does afflict every geography in the world, it is also evident that Eastern and Central European countries have a particularly high burden, which may be related to higher risk of falls in those countries as described below. We also identified regions where falls were not the leading cause, such as Oceania, where physical violence by other means predominated in males. This finding may be related to the relatively higher incidence of physical violence by other means in Oceania and Southern sub-Saharan Africa in GBD 2017.

The second overarching theme is that falls are the predominant cause of facial fractures, which is consistent with our clinical experiences at level 1 trauma centres in the USA. While falls are not frequently considered global health priorities, they nevertheless inflict considerable disability in multiple populations around the globe and have persisted as a high-ranking cause of YLDs in the GBD.^34^ This study highlights the disabling effects falls can have, specifically when they result in a condition that requires a higher level of care and subspecialised intervention. The potential complexity of these injuries is a compelling argument for prevention strategies focused on mitigating fall risk. The factors that can prevent such injuries from occurring likely depend largely on geographical and age-related factors. In young age groups, the risk of falls may be related to the built environment,^35–37^ income,^35^ furniture,^38^ or other factors. Some falls in this population may be averted through educational programme and ensuring safe conditions early in life.^39 40^ In adult populations, according to research that did not include the elderly, alcohol use appears to be one of the prominent risk factors associated with falls.^41^ In elderly populations, in which there is an increased incidence of falls with increasing age,^34^ the incidence of falls may also be driven by medication use, vision impairment, frailty, alcohol abuse and environmental factors.^31 33 42 43^ A disabling injury such as a facial fracture is detrimental to one’s functional status and can be costly both for the individual and the healthcare system.^44–46^ Hence, addressing the factors that lead to falls may be one of the most tractable methods for preventing facial fractures in this population. We also observed that while falls were the predominant cause of facial fractures, there were other critical causes, in particular related to physical violence by other means and other exposure to mechanical forces.

The third main finding is that the North Africa and Middle East region stands out by being the only region where facial fractures were not predominantly driven by falls in 2017. Instead, the burden was most heavily driven by conflict and terrorism. Since war can have significantly detrimental impacts on a country’s healthcare system and impair the population’s ability to access and receive medical and surgical services, the victims of facial fractures due to conflict and terrorism in North Africa and the Middle East likely lack proper access to the surgical and medical services that would help mitigate the disability and disfigurement from these injuries. Furthermore, these injuries are more likely to be secondary to high-energy mechanism injuries (eg, high-velocity blunt force trauma, shrapnel and ballistic injuries). These mechanisms more frequently result in operative facial fracture patterns with varying degrees of soft-tissue, ocular and nerve injury, based on our clinical experience. Since improperly treated facial fractures, especially in this setting, can cause considerable long-term disability and disfigurement, the victims of these war-time injuries may experience lifelong sequelae of their facial trauma. Other violent aetiologies of facial fractures, such as physical violence by other means (which is the interpersonal violence subcause in the GBD hierarchy that excludes violence with firearms, sharp objects and sexual violence), also appear as significant contributors to the burden of facial fractures in this study, and indicate how violent behaviour such as domestic abuse and other assault that don’t involve weapons are important drivers of facial fractures.

The current study has several limitations. First, since our estimation of facial fractures depends on the GBD 2017 estimates for all external causes of injury, the limitations in terms of data coverage and modelling processes that are described in other GBD literature also apply here.^17^ The limitations of data coverage are particularly pertinent to lower income areas in which the GBD has limited amounts of the clinical and hospital data that are used heavily in injuries estimation, so models must rely more heavily on covariates in these locations. Second, our method for estimating the cause-nature relationships of injuries to facial fractures depends on dual-coded hospital data, which is not available in every country with hospital data and therefore represents a limited subset of all areas included in the GBD location hierarchy. It would improve our estimation process to have more dual-coded hospital data in our estimation process, and in future iterations of the GBD, we plan to continue adding such datasets to our clinical database. Third, due to data constraints in GBD 2017, we were unable to separately estimate disability weights for treated and untreated facial fractures (regardless of whether ‘treated’ status refers to non-operative care or to a form of reduction with or without rigid fixation). This limitation has likely impacted the geographic heterogeneity of our facial fracture YLD estimates since higher income locations likely have higher rates of treatment than lower income locations, though it does not impact the incidence and prevalence estimation processes. Finally, as noted in the methods section above, the study design employs an assumption that injury disability is determined by the most severe nature of injury sustained for a given cause of injury. As such, in the instances where an individual sustains both a facial fracture and a more disabling injury such as a spinal cord or closed head injury in the dual-coded proportion split process, facial fractures go uncounted in the process where the per cent of a given cause that lead to facial fractures are estimated. As a result, it is likely that a number of facial fractures are missed as being the most severe injury sustained. In addition, mechanistically, since the face acts as an air-filled network of bones and sinuses that decelerate the head and cushion the neurological structures behind them, there is likely considerable risk of concomitant intracranial and cervical spine injuries occurring in the event of facial bone trauma.^14 47^ Future iterations of the GBD could address this limitation by modelling and estimating both cause of injury and nature of injury as separate entities, since we would not need to make the assumption about hierarchical severities determining disability.

### Conclusion

Facial fractures have various causes and occur within every population in the world, though select locations currently experience a higher burden. Facial fractures are predominantly driven by falls except in regions suffering from conflict. Given that surgical treatment of facial fractures can require considerable expertise and that the disability experienced with facial fractures may be mitigated with such treatment, it is important for healthcare systems around the world to develop injury prevention programme and to ensure that individuals who experience facial fractures have adequate access to care and treatment. In addition, this study emphasises the need for more expansive data collection and utilisation where both cause and nature of injury can be identified.

**What is already known on this subject**

- Facial fractures are disabling injuries that can occur as the result of various causes of injury.
- Facial fractures are known to occur globally, but resulting disability can be affected by the availability of surgical treatment and by the severity of injury.

**What this study adds**

- Falls are the leading cause of facial fractures globally.
- Facial fractures are most concentrated in Central Europe.
- In 2017, there were an estimated 7.5 million new cases of facial fractures with 1.8 million individuals living with disability from a facial fracture.
