# Correction: *Epidemiology of facial fractures: incidence, prevalence and years lived with disability estimates from the Global Burden of Disease 2017 study*

**DOI:** 10.1136/injuryprev-2019-043297corr1

**Published:** 2022-12-07

Lalloo R, Lucchesi LR, Bisignano C, *et al*. Epidemiology of facial fractures: incidence, prevalence and years lived with disability estimates from the Global Burden of Disease 2017 study. *Inj Prev* 2020;26:i27–i35. doi: 10.1136/injuryprev-2019-043297

The author Navid Manafi’s surname was incorrectly spelt as 'Manaf'.

