# Supplementary material for: Epidemiology of facial fractures: incidence, prevalence and years lived with disability estimates from the Global Burden of Disease 2017 study
Source: Inj Prev. 2020 Jan 8;26(Suppl 1):i27–35. doi: 10.1136/injuryprev-2019-043297 (PMC7571355; doi:10.1136/injuryprev-2019-043297)
Supplement: Supplementary data [file injuryprev-2019-043297supp003.pdf]

Table 1: Incidence, prevalence, and YLDs for 2017 and percentage change of age-standardised rates by location for facial fractures

Location	Incidence (95% UI)			Prevalence (95% UI)			YLDs (95% UI)		
	2017 counts	2017 age-standardised rates per 100,000	Percentage change in age-standardised rates between 1990 and 2017	2017 counts	2017 age-standardised rates per 100,000	Percentage change in age-standardised rates between 1990 and 2017	2017 counts	2017 age-standardised rates per 100,000	Percentage change in age-standardised rates between 1990 and 2017
**Global**	**7 538 663 (6 116 489 to 9 493 113)**	**98 (80 to 123)**	**-2.0 (-3.9 to 0.2)**	**1 819 732 (1 609 419 to 2 091 618)**	**23 (20 to 27)**	**-2.8 (-4.1 to -1.4)**	**117 402 (73 266 to 169 689)**	**1 (1 to 2)**	**-2.7 (-4.3 to -1.0)**
**Low SDI**	**1 089 162 (886 300 to 1 327 015)**	**86 (70 to 105)**	**-12.3 (-28.2 to -0.3)**	**219 785 (184 916 to 270 384)**	**21 (18 to 25)**	**-1.8 (-12.0 to 4.6)**	**14 292 (9 003 to 20 425)**	**1 (1 to 2)**	**-2.2 (-12.7 to 5.5)**
**Low-middle SDI**	**1 456 718 (1 200 207 to 1 777 972)**	**86 (70 to 104)**	**17.3 (13.4 to 21.6)**	**300 831 (259 456 to 352 338)**	**19 (17 to 23)**	**15.7 (13.5 to 18.0)**	**19 567 (12 174 to 28 035)**	**1 (1 to 2)**	**15.7 (12.1 to 19.2)**
**Middle SDI**	**1 511 639 (1 213 269 to 1 875 589)**	**72 (58 to 89)**	**24.3 (18.8 to 30.8)**	**364 245 (322 625 to 418 366)**	**17 (15 to 19)**	**22.4 (19.2 to 26.5)**	**23 653 (14 840 to 34 586)**	**1 (1 to 2)**	**22.2 (18.3 to 26.9)**
**High-middle SDI**	**1 749 211 (1 386 163 to 2 235 915)**	**128 (102 to 164)**	**0.4 (-2.6 to 3.5)**	**453 558 (402 694 to 517 635)**	**29 (26 to 34)**	**-3.1 (-4.6 to -1.5)**	**29 163 (18 075 to 42 458)**	**2 (1 to 3)**	**-2.8 (-5.2 to -0.2)**
**High SDI**	**1 709 496 (1 307 361 to 2 264 741)**	**158 (122 to 206)**	**-10.3 (-12.4 to -8.1)**	**475 199 (420 916 to 544 481)**	**35 (30 to 41)**	**-9.6 (-10.8 to -8.4)**	**30 332 (18 841 to 44 614)**	**2 (1 to 3)**	**-9.7 (-11.7 to -7.7)**
**Central Europe, Eastern Europe, and Central Asia**	**1 024 479 (774 842 to 1 360 176)**	**254 (193 to 335)**	**-5.4 (-8.2 to -2.5)**	**265 683 (232 728 to 309 889)**	**56 (48 to 67)**	**-4.9 (-6.5 to -3.1)**	**16 947 (10 461 to 24 821)**	**4 (2 to 5)**	**-4.9 (-7.3 to -2.4)**
**Central Asia**	**154 199 (120 104 to 199 933)**	**167 (130 to 217)**	**-3.4 (-7.5 to 0.6)**	**33 550 (28 926 to 39 751)**	**38 (33 to 44)**	**-2.5 (-5.1 to 0.9)**	**2 176 (1 332 to 3 192)**	**2 (1 to 4)**	**-2.5 (-7.6 to 3.3)**
Armenia	4 734 (3 647 to 6 235)	163 (125 to 214)	-22.7 (-32.5 to -15.6)	1 235 (1 072 to 1 435)	37 (32 to 44)	-21.9 (-27.7 to -17.5)	79 (49 to 117)	2 (1 to 4)	-21.8 (-31.6 to -11.6)
Azerbaijan	17 127 (13 275 to 22 375)	166 (128 to 215)	0.4 (-7.9 to 7.3)	3 964 (3 427 to 4 683)	38 (33 to 45)	4.6 (-0.8 to 12.2)	256 (162 to 377)	2 (2 to 4)	4.4 (-6.9 to 18.1)
Georgia	6 094 (4 723 to 7 897)	171 (133 to 220)	-3.9 (-10.1 to 2.9)	1 627 (1 435 to 1 871)	38 (33 to 45)	-2.1 (-6.3 to 2.9)	104 (64 to 152)	2 (2 to 4)	-2.4 (-11.3 to 8.6)
Kazakhstan	34 224 (26 818 to 43 925)	191 (149 to 246)	-2.3 (-7.0 to 2.6)	7 567 (6 554 to 8 897)	42 (36 to 49)	-2.9 (-5.7 to 0.1)	489 (300 to 730)	3 (2 to 4)	-2.7 (-12.9 to 8.4)
Kyrgyzstan	9 831 (7 635 to 12 776)	150 (117 to 196)	-19.5 (-25.1 to -14.3)	2 020 (1 718 to 2 414)	34 (29 to 40)	-18.6 (-21.9 to -15.7)	132 (79 to 199)	2 (1 to 3)	-18.4 (-27.4 to -8.1)
Mongolia	6 683 (5 137 to 8 665)	200 (154 to 260)	17.3 (12.9 to 21.9)	1 397 (1 197 to 1 654)	44 (38 to 52)	13.9 (10.7 to 16.9)	91 (55 to 136)	3 (2 to 4)	14.0 (2.6 to 27.6)
Tajikistan	14 906 (11 428 to 19 539)	156 (119 to 204)	-4.1 (-9.2 to 0.8)	3 189 (2 646 to 3 950)	39 (32 to 47)	5.4 (-3.9 to 26.3)	207 (128 to 309)	2 (2 to 4)	4.9 (-8.7 to 29.0)
Turkmenistan	8 405 (6 489 to 10 855)	166 (128 to 216)	9.3 (3.6 to 15.0)	1 772 (1 520 to 2 104)	37 (31 to 43)	6.6 (3.3 to 9.7)	115 (70 to 173)	2 (1 to 4)	6.6 (-4.9 to 20.1)
Uzbekistan	52 196 (40 394 to 67 877)	157 (122 to 204)	2.8 (-2.1 to 7.7)	10 777 (9 222 to 12 843)	35 (30 to 41)	2.8 (-0.1 to 5.7)	702 (421 to 1 047)	2 (1 to 3)	2.8 (-7.4 to 14.3)
**Central Europe**	**337 910 (245 337 to 464 014)**	**310 (228 to 420)**	**-1.8 (-6.0 to 2.1)**	**92 387 (80 541 to 108 397)**	**68 (57 to 82)**	**-2.9 (-5.2 to -0.5)**	**5 861 (3 621 to 8 694)**	**4 (3 to 7)**	**-2.8 (-6.8 to 1.3)**
Albania	7 390 (5 473 to 9 981)	281 (208 to 377)	10.1 (2.4 to 17.3)	1 897 (1 649 to 2 240)	62 (52 to 74)	9.5 (4.8 to 14.3)	122 (74 to 182)	4 (2 to 6)	9.5 (-0.3 to 20.8)
Bosnia and Herzegovina	8 812 (6 479 to 12 000)	286 (211 to 386)	29.5 (25.0 to 34.4)	2 686 (2 294 to 3 257)	68 (57 to 83)	38.7 (29.0 to 60.4)	170 (108 to 242)	4 (3 to 6)	38.1 (22.7 to 65.3)
Bulgaria	18 901 (13 796 to 25 776)	295 (218 to 396)	-4.2 (-9.4 to 1.2)	5 452 (4 777 to 6 356)	64 (54 to 77)	-4.1 (-7.4 to -0.5)	346 (214 to 514)	4 (2 to 6)	-4.0 (-12.0 to 4.3)
Croatia	10 801 (7 838 to 14 942)	248 (187 to 331)	-16.5 (-22.7 to -10.4)	3 017 (2 630 to 3 542)	55 (47 to 66)	-13.5 (-17.6 to -8.6)	191 (118 to 282)	4 (2 to 5)	-13.5 (-21.7 to -4.5)
Czech Republic	36 609 (26 366 to 49 959)	374 (275 to 500)	4.2 (-2.5 to 11.1)	10 166 (8 837 to 12 000)	81 (69 to 99)	3.8 (-0.1 to 8.2)	642 (391 to 958)	5 (3 to 8)	3.8 (-4.3 to 14.1)
Hungary	28 311 (20 302 to 39 443)	303 (220 to 413)	-10.2 (-16.1 to -4.1)	7 785 (6 705 to 9 226)	66 (55 to 80)	-12.2 (-15.8 to -8.5)	493 (298 to 742)	4 (3 to 6)	-11.6 (-19.0 to -3.0)
Macedonia	5 698 (4 197 to 7 813)	282 (208 to 382)	10.2 (0.6 to 18.0)	1 500 (1 300 to 1 772)	62 (52 to 75)	12.4 (7.7 to 16.8)	96 (59 to 144)	4 (2 to 6)	12.3 (1.5 to 23.2)
Montenegro	1 759 (1 306 to 2 362)	298 (222 to 402)	3.6 (-2.0 to 9.0)	455 (394 to 533)	65 (55 to 78)	4.5 (1.0 to 8.4)	29 (18 to 43)	4 (3 to 6)	4.3 (-5.2 to 14.3)
Poland	116 518 (84 517 to 161 202)	316 (233 to 427)	1.5 (-3.5 to 6.9)	31 345 (27 039 to 36 935)	69 (58 to 83)	-0.4 (-3.7 to 2.8)	1 991 (1 228 to 2 954)	4 (3 to 7)	-0.2 (-8.7 to 8.5)
Romania	55 334 (40 328 to 75 628)	300 (220 to 407)	-12.4 (-18.0 to -6.4)	15 183 (13 164 to 17 819)	65 (55 to 79)	-14.0 (-17.2 to -10.5)	963 (582 to 1 428)	4 (3 to 6)	-13.9 (-21.4 to -6.1)
Serbia	23 331 (17 109 to 31 741)	284 (210 to 383)	10.1 (4.2 to 15.9)	6 397 (5 559 to 7 558)	63 (53 to 76)	10.0 (5.9 to 15.0)	406 (249 to 594)	4 (2 to 6)	9.9 (0.3 to 21.1)
Slovakia	16 811 (12 185 to 23 128)	320 (235 to 430)	-7.0 (-11.8 to -2.4)	4 401 (3 789 to 5 199)	69 (58 to 84)	-8.5 (-11.4 to -5.5)	280 (168 to 417)	4 (3 to 7)	-8.2 (-15.3 to -0.6)
Slovenia	7 634 (5 419 to 10 622)	376 (272 to 507)	0.3 (-5.4 to 6.6)	2 105 (1 829 to 2 484)	81 (69 to 99)	-0.8 (-4.2 to 3.0)	133 (81 to 199)	5 (3 to 8)	-0.7 (-8.6 to 8.0)
**Eastern Europe**	**532 370 (407 819 to 700 276)**	**268 (205 to 351)**	**-2.3 (-5.2 to 1.0)**	**139 745 (122 815 to 161 152)**	**58 (50 to 69)**	**-2.3 (-4.1 to -0.4)**	**8 911 (5 526 to 13 012)**	**4 (2 to 6)**	**-2.3 (-5.4 to 0.9)**
Belarus	23 553 (17 817 to 31 184)	262 (198 to 344)	2.0 (-3.0 to 6.8)	6 181 (5 380 to 7 219)	56 (48 to 67)	0.7 (-2.1 to 3.7)	395 (242 to 579)	4 (2 to 5)	0.9 (-8.0 to 11.0)
Estonia	3 101 (2 357 to 4 110)	257 (195 to 336)	-16.9 (-22.0 to -11.7)	852 (748 to 991)	55 (47 to 66)	-17.3 (-20.3 to -14.1)	54 (33 to 81)	4 (2 to 5)	-17.1 (-24.7 to -9.4)
Latvia	4 778 (3 612 to 6 356)	263 (199 to 346)	-17.1 (-22.1 to -12.1)	1 324 (1 164 to 1 528)	56 (48 to 67)	-18.4 (-21.3 to -15.3)	84 (52 to 125)	4 (2 to 5)	-18.2 (-24.9 to -11.3)
Lithuania	7 612 (5 696 to 10 252)	277 (210 to 362)	-6.2 (-11.0 to -1.1)	2 091 (1 826 to 2 431)	60 (51 to 71)	-7.0 (-9.9 to -3.8)	132 (82 to 194)	4 (2 to 6)	-7.0 (-14.8 to 1.1)
Moldova	7 737 (5 897 to 10 158)	224 (170 to 293)	-12.7 (-17.6 to -7.3)	2 032 (1 772 to 2 361)	49 (42 to 58)	-13.7 (-16.8 to -10.4)	130 (80 to 193)	3 (2 to 5)	-13.3 (-22.2 to -4.3)
Russian Federation	377 124 (289 211 to 496 594)	271 (207 to 354)	-3.2 (-7.3 to 1.1)	98 094 (86 163 to 113 401)	59 (50 to 70)	-2.8 (-5.2 to -0.4)	6 259 (3 864 to 9 187)	4 (2 to 6)	-2.8 (-6.1 to 0.5)
Ukraine	108 465 (83 222 to 142 677)	264 (202 to 346)	2.0 (-2.2 to 6.4)	29 173 (25 707 to 33 553)	57 (49 to 68)	1.2 (-1.6 to 4.1)	1 856 (1 138 to 2 740)	4 (2 to 5)	1.0 (-7.2 to 10.5)
**High-income**	**1 530 492 (1 180 646 to 2 011 370)**	**150 (117 to 195)**	**-10.8 (-13.0 to -8.5)**	**420 726 (373 075 to 481 966)**	**33 (28 to 39)**	**-10.4 (-11.6 to -9.1)**	**26 878 (16 711 to 39 478)**	**2 (1 to 3)**	**-10.5 (-12.6 to -8.4)**
**Australasia**	**77 125 (58 731 to 101 370)**	**291 (222 to 381)**	**10.1 (5.6 to 14.7)**	**19 522 (17 023 to 22 762)**	**62 (53 to 73)**	**9.5 (6.4 to 12.8)**	**1 250 (773 to 1 852)**	**4 (2 to 6)**	**9.4 (1.4 to 18.6)**
Australia	62 547 (47 525 to 82 203)	281 (214 to 366)	10.1 (4.9 to 15.1)	15 858 (13 802 to 18 496)	60 (51 to 71)	9.7 (6.2 to 13.3)	1 016 (627 to 1 508)	4 (2 to 6)	9.7 (0.1 to 20.7)
New Zealand	14 579 (11 142 to 19 157)	345 (266 to 449)	11.8 (6.2 to 18.3)	3 663 (3 195 to 4 278)	73 (63 to 87)	10.0 (6.0 to 14.4)	234 (144 to 347)	5 (3 to 7)	10.0 (1.3 to 19.1)
**High-income Asia-Pacific**	**259 948 (194 962 to 350 170)**	**158 (119 to 214)**	**4.7 (0.3 to 8.9)**	**80 146 (70 927 to 91 929)**	**35 (30 to 42)**	**5.6 (3.3 to 7.9)**	**5 112 (3 201 to 7 511)**	**2 (1 to 3)**	**5.6 (1.1 to 10.2)**
Brunei	812 (635 to 1 046)	190 (149 to 246)	-0.2 (-4.9 to 4.1)	172 (148 to 204)	41 (35 to 48)	-3.2 (-6.0 to -0.4)	11 (7 to 17)	3 (2 to 4)	-2.8 (-13.1 to 8.2)
Japan	174 530 (126 189 to 241 959)	155 (114 to 215)	16.7 (12.4 to 20.6)	57 496 (50 719 to 66 017)	35 (30 to 42)	16.7 (14.0 to 19.2)	3 653 (2 280 to 5 403)	2 (1 to 3)	16.8 (12.6 to 20.9)
South Korea	76 028 (59 055 to 99 388)	163 (126 to 211)	-15.2 (-20.1 to -10.3)	20 334 (17 868 to 23 353)	35 (30 to 41)	-17.0 (-19.9 to -14.2)	1 308 (798 to 1 912)	2 (1 to 3)	-16.8 (-25.0 to -7.4)
Singapore	8 578 (6 664 to 11 129)	169 (131 to 220)	1.3 (-2.6 to 5.4)	2 144 (1 871 to 2 488)	36 (31 to 43)	3.0 (0.3 to 5.8)	139 (86 to 207)	2 (1 to 4)	2.9 (-8.1 to 15.2)
**High-income North America**	**481 467 (361 683 to 642 026)**	**131 (101 to 170)**	**-30.6 (-34.2 to -27.1)**	**132 599 (117 213 to 151 321)**	**30 (27 to 35)**	**-29.2 (-31.4 to -27.0)**	**8 451 (5 258 to 12 337)**	**2 (1 to 3)**	**-29.4 (-32.4 to -26.3)**
Canada	49 267 (38 614 to 63 397)	143 (113 to 181)	-0.5 (-4.2 to 3.4)	13 435 (11 901 to 15 335)	31 (27 to 36)	-1.0 (-3.4 to 1.2)	863 (530 to 1 269)	2 (1 to 3)	-1.0 (-10.1 to 9.2)
Greenland	87 (68 to 112)	154 (121 to 197)	-33.2 (-36.6 to -30.3)	21 (18 to 24)	34 (30 to 40)	-34.7 (-36.8 to -32.7)	1 (1 to 2)	2 (1 to 3)	-34.6 (-41.2 to -27.4)
USA	432 104 (322 525 to 576 650)	130 (99 to 170)	-32.8 (-36.7 to -29.1)	119 141 (105 314 to 136 479)	30 (27 to 35)	-31.2 (-33.6 to -28.9)	7 586 (4 709 to 11 040)	2 (1 to 3)	-31.5 (-34.6 to -28.3)
**Southern Latin America**	**95 910 (76 815 to 122 752)**	**149 (120 to 191)**	**13.8 (8.5 to 19.7)**	**22 100 (19 379 to 25 409)**	**32 (28 to 37)**	**10.7 (7.5 to 14.0)**	**1 428 (887 to 2 089)**	**2 (1 to 3)**	**10.5 (1.7 to 20.8)**
Argentina	66 405 (52 911 to 84 872)	152 (121 to 194)	18.0 (12.1 to 24.8)	15 189 (13 284 to 17 529)	33 (29 to 38)	15.0 (11.5 to 19.0)	982 (612 to 1 444)	2 (1 to 3)	14.8 (2.8 to 28.5)
Chile	24 217 (19 347 to 30 820)	138 (110 to 175)	3.2 (-4.6 to 10.6)	5 612 (4 931 to 6 479)	29 (25 to 34)	-0.2 (-4.5 to 4.0)	363 (223 to 531)	2 (1 to 3)	-0.3 (-11.9 to 12.4)
Uruguay	5 283 (4 170 to 6 836)	159 (126 to 205)	13.7 (7.9 to 20.1)	1 297 (1 141 to 1 487)	34 (30 to 40)	11.9 (8.3 to 15.5)	84 (52 to 123)	2 (1 to 3)	12.1 (1.3 to 26.0)
**Western Europe**	**616 043 (474 017 to 813 742)**	**155 (120 to 202)**	**-2.9 (-5.8 to -0.2)**	**166 361 (145 619 to 192 115)**	**33 (28 to 39)**	**-3.3 (-5.0 to -1.7)**	**10 637 (6 602 to 15 577)**	**2 (1 to 3)**	**-3.3 (-6.9 to 0.1)**
Andorra	118 (91 to 156)	165 (127 to 215)	1.6 (-1.8 to 4.9)	32 (28 to 37)	35 (30 to 41)	1.5 (-0.5 to 3.7)	2 (1 to 3)	2 (1 to 3)	1.2 (-8.8 to 13.0)
Austria	13 112 (10 096 to 17 329)	163 (125 to 211)	-10.9 (-15.2 to -6.2)	3 537 (3 094 to 4 104)	34 (29 to 41)	-11.3 (-13.8 to -8.8)	226 (138 to 332)	2 (1 to 3)	-11.4 (-20.1 to -1.8)
Belgium	19 363 (14 793 to 25 487)	179 (138 to 234)	8.3 (3.8 to 13.1)	5 067 (4 403 to 5 909)	38 (32 to 45)	6.5 (3.5 to 9.6)	323 (200 to 479)	2 (1 to 4)	6.6 (-4.7 to 18.3)
Cyprus	1 894 (1 474 to 2 441)	163 (127 to 210)	-2.0 (-5.8 to 1.9)	476 (417 to 550)	34 (29 to 41)	-4.7 (-7.4 to -2.1)	31 (19 to 45)	2 (1 to 3)	-4.4 (-14.7 to 6.5)
Denmark	8 270 (6 353 to 10 854)	159 (123 to 206)	-3.5 (-7.4 to 0.4)	2 183 (1 907 to 2 530)	33 (28 to 40)	-3.2 (-5.9 to -0.4)	140 (86 to 205)	2 (1 to 3)	-3.0 (-12.0 to 8.2)
Finland	9 791 (7 437 to 13 057)	188 (145 to 245)	1.7 (-2.1 to 6.0)	2 607 (2 277 to 3 036)	39 (33 to 47)	2.2 (-0.3 to 4.7)	166 (102 to 245)	3 (2 to 4)	2.3 (-7.6 to 13.5)
France	103 333 (79 304 to 136 871)	166 (128 to 216)	-5.3 (-9.2 to -1.7)	27 149 (23 707 to 31 425)	35 (30 to 41)	-6.0 (-8.4 to -3.7)	1 740 (1 060 to 2 569)	2 (1 to 3)	-5.9 (-15.6 to 4.0)
Germany	119 967 (91 450 to 159 297)	159 (122 to 207)	1.7 (-2.2 to 5.6)	33 493 (29 396 to 38 795)	33 (28 to 40)	1.0 (-1.9 to 3.6)	2 137 (1 313 to 3 131)	2 (1 to 3)	1.1 (-8.7 to 11.7)
Greece	14 251 (11 043 to 18 542)	157 (123 to 203)	-2.9 (-6.7 to 0.6)	4 085 (3 620 to 4 680)	33 (29 to 40)	-3.0 (-5.4 to -0.5)	260 (159 to 382)	2 (1 to 3)	-3.2 (-14.1 to 8.3)
Iceland	510 (395 to 663)	160 (125 to 208)	2.5 (-1.1 to 6.1)	125 (108 to 146)	34 (29 to 40)	1.5 (-0.6 to 4.1)	8 (5 to 12)	2 (1 to 3)	1.4 (-9.4 to 14.5)
Ireland	7 077 (5 452 to 9 232)	157 (121 to 205)	3.1 (-0.5 to 6.7)	1 725 (1 498 to 2 012)	33 (28 to 39)	2.6 (0.1 to 5.2)	111 (67 to 164)	2 (1 to 3)	2.5 (-8.4 to 14.1)
Israel	13 578 (10 495 to 17 550)	155 (120 to 200)	0.7 (-5.6 to 5.6)	3 109 (2 663 to 3 673)	34 (29 to 40)	6.1 (1.2 to 13.4)	201 (124 to 294)	2 (1 to 3)	6.0 (-6.4 to 21.0)
Italy	77 022 (58 634 to 101 556)	138 (107 to 180)	-13.0 (-16.5 to -9.7)	21 913 (19 264 to 25 209)	29 (25 to 34)	-13.3 (-15.5 to -11.0)	1 398 (865 to 2 060)	2 (1 to 3)	-13.4 (-21.6 to -4.6)
Luxembourg	939 (726 to 1 234)	169 (131 to 220)	-16.9 (-21.5 to -12.3)	239 (209 to 277)	36 (30 to 42)	-14.4 (-17.0 to -11.7)	15 (9 to 23)	2 (1 to 3)	-14.5 (-23.2 to -4.5)
Malta	659 (499 to 869)	170 (131 to 221)	3.5 (-1.1 to 8.2)	177 (155 to 206)	35 (30 to 42)	5.6 (2.6 to 8.5)	11 (7 to 17)	2 (1 to 3)	5.8 (-4.5 to 17.3)
Netherlands	21 351 (16 375 to 28 253)	137 (107 to 176)	3.0 (-2.7 to 8.2)	5 542 (4 867 to 6 402)	28 (24 to 33)	0.9 (-2.3 to 4.1)	355 (219 to 516)	2 (1 to 3)	1.0 (-10.2 to 13.3)
Norway	7 732 (5 558 to 10 734)	154 (112 to 214)	-2.1 (-4.9 to 0.6)	2 104 (1 828 to 2 486)	34 (29 to 42)	-1.7 (-3.2 to -0.1)	134 (82 to 200)	2 (1 to 3)	-1.7 (-5.2 to 1.7)
Portugal	12 649 (9 806 to 16 515)	132 (104 to 170)	-25.1 (-29.5 to -20.7)	3 544 (3 130 to 4 078)	28 (24 to 33)	-26.0 (-28.7 to -23.3)	226 (136 to 334)	2 (1 to 3)	-26.0 (-34.1 to -16.5)
Spain	61 719 (47 790 to 80 736)	150 (116 to 195)	-6.0 (-10.0 to -2.0)	17 066 (15 010 to 19 557)	32 (27 to 38)	-6.3 (-8.5 to -3.9)	1 092 (672 to 1 604)	2 (1 to 3)	-6.3 (-16.0 to 4.7)
Sweden	13 904 (9 877 to 19 514)	146 (106 to 204)	7.8 (4.5 to 11.3)	3 940 (3 420 to 4 617)	33 (28 to 40)	7.8 (5.5 to 10.6)	252 (157 to 379)	2 (1 to 3)	7.9 (-2.6 to 18.7)
Switzerland	13 030 (9 896 to 17 419)	158 (122 to 208)	-25.5 (-29.5 to -21.0)	3 469 (3 042 to 4 013)	33 (29 to 40)	-24.1 (-26.5 to -21.4)	222 (135 to 329)	2 (1 to 3)	-24.0 (-30.9 to -15.8)

Location	Incidence (95% UI)			Prevalence (95% UI)			YLDs (95% UI)		
	2017 counts	2017 age-standardised rates per 100,000	Percentage change in age-standardised rates between 1990 and 2017	2017 counts	2017 age-standardised rates per 100,000	Percentage change in age-standardised rates between 1990 and 2017	2017 counts	2017 age-standardised rates per 100,000	Percentage change in age-standardised rates between 1990 and 2017
United Kingdom	95 135 (73 259 to 125 133)	156 (120 to 203)	8.7 (5.9 to 11.5)	24 606 (21 455 to 28 573)	33 (28 to 39)	9.1 (7.3 to 11.0)	1 575 (973 to 2 307)	2 (1 to 3)	9.0 (6.2 to 11.8)
**Latin America and Caribbean**	**408 817 (312 608 to 535 700)**	**70 (54 to 92)**	**9.3 (-0.7 to 17.5)**	**94 277 (81 944 to 111 340)**	**16 (14 to 19)**	**10.3 (3.9 to 15.4)**	**6 119 (3 803 to 9 103)**	**1 (1 to 2)**	**10.1 (3.4 to 16.3)**
**Andean Latin America**	**38 201 (30 281 to 48 250)**	**63 (50 to 79)**	**-15.1 (-39.3 to 3.9)**	**8 463 (7 330 to 9 954)**	**14 (12 to 17)**	**-6.4 (-21.6 to 4.2)**	**551 (341 to 804)**	**1 (1 to 1)**	**-6.6 (-23.4 to 7.9)**
Bolivia	6 615 (5 232 to 8 393)	59 (46 to 74)	3.8 (-0.8 to 9.0)	1 357 (1 167 to 1 596)	13 (11 to 15)	1.8 (-0.9 to 4.8)	89 (53 to 130)	1 (1 to 1)	1.7 (-9.8 to 14.4)
Ecuador	11 444 (9 107 to 14 542)	69 (55 to 88)	14.7 (10.0 to 19.6)	2 471 (2 133 to 2 901)	15 (13 to 18)	9.5 (5.6 to 12.9)	161 (100 to 238)	1 (1 to 1)	9.8 (-4.0 to 24.2)
Peru	20 141 (15 991 to 25 494)	61 (48 to 77)	-28.5 (-55.5 to -2.1)	4 635 (3 954 to 5 541)	14 (12 to 17)	-14.3 (-34.4 to 1.9)	302 (185 to 440)	1 (1 to 1)	-14.6 (-36.2 to 7.6)
**Caribbean**	**33 496 (26 849 to 41 646)**	**72 (58 to 90)**	**36.3 (28.5 to 52.0)**	**8 430 (6 982 to 10 524)**	**18 (14 to 22)**	**50.7 (33.0 to 84.0)**	**543 (344 to 782)**	**1 (1 to 2)**	**48.8 (29.8 to 84.3)**
Antigua and Barbuda	59 (47 to 75)	68 (55 to 87)	32.1 (27.2 to 38.1)	14 (12 to 16)	14 (13 to 17)	27.6 (24.3 to 31.2)	1 (1 to 1)	1 (1 to 1)	27.3 (13.6 to 42.9)
The Bahamas	245 (198 to 306)	67 (54 to 83)	28.1 (23.4 to 33.2)	55 (48 to 63)	14 (12 to 17)	25.8 (22.7 to 29.3)	4 (2 to 5)	1 (1 to 1)	25.4 (11.1 to 42.0)
Barbados	179 (142 to 226)	63 (50 to 78)	34.1 (29.6 to 38.6)	45 (40 to 52)	13 (11 to 15)	32.1 (29.2 to 34.8)	3 (2 to 4)	1 (1 to 1)	31.5 (17.2 to 47.6)
Belize	269 (219 to 335)	69 (56 to 86)	43.8 (38.1 to 49.3)	56 (48 to 65)	16 (14 to 18)	46.7 (41.7 to 54.5)	4 (2 to 5)	1 (1 to 1)	45.5 (27.4 to 67.5)
Bermuda	44 (34 to 56)	68 (54 to 84)	27.0 (22.1 to 31.6)	12 (11 to 14)	15 (13 to 17)	20.8 (13.5 to 25.3)	1 (0 to 1)	1 (1 to 1)	21.1 (4.7 to 37.9)
Cuba	8 317 (6 399 to 10 908)	70 (55 to 90)	24.7 (19.3 to 30.0)	2 032 (1 776 to 2 372)	15 (13 to 17)	20.7 (17.3 to 24.2)	131 (80 to 193)	1 (1 to 1)	20.5 (7.0 to 35.4)
Dominica	42 (33 to 52)	61 (49 to 76)	41.8 (37.0 to 46.5)	10 (9 to 12)	13 (12 to 15)	41.2 (36.7 to 45.0)	1 (0 to 1)	1 (1 to 1)	40.4 (23.5 to 59.6)
Dominican Republic	7 644 (6 224 to 9 472)	73 (60 to 91)	52.3 (46.4 to 58.3)	1 617 (1 405 to 1 873)	16 (14 to 18)	48.7 (44.9 to 52.2)	106 (65 to 156)	1 (1 to 2)	47.6 (29.4 to 67.6)
Grenada	70 (56 to 89)	63 (51 to 80)	31.9 (27.4 to 36.7)	17 (15 to 19)	14 (12 to 16)	29.7 (26.3 to 32.9)	1 (1 to 2)	1 (1 to 1)	29.1 (15.3 to 45.1)
Guyana	474 (381 to 589)	65 (52 to 81)	34.7 (29.6 to 39.7)	100 (87 to 117)	14 (12 to 16)	33.2 (30.1 to 36.7)	7 (4 to 10)	1 (1 to 1)	32.5 (17.5 to 48.9)
Haiti	6 116 (4 946 to 7 567)	53 (43 to 66)	5.3 (-0.7 to 10.9)	2 368 (1 474 to 4 052)	24 (15 to 41)	105.7 (29.8 to 261.6)	149 (85 to 264)	1 (1 to 3)	97.5 (25.4 to 257.4)
Jamaica	1 977 (1 592 to 2 492)	71 (58 to 90)	49.8 (42.0 to 58.4)	437 (382 to 507)	15 (13 to 18)	48.8 (43.8 to 53.5)	29 (18 to 42)	1 (1 to 1)	48.1 (30.0 to 69.1)
Puerto Rico	5 245 (3 382 to 8 556)	148 (94 to 248)	152.0 (71.4 to 337.1)	985 (777 to 1 360)	24 (18 to 34)	86.9 (47.7 to 170.6)	64 (36 to 102)	2 (1 to 3)	88.4 (43.5 to 180.0)
Saint Lucia	109 (88 to 136)	63 (51 to 79)	31.8 (27.3 to 36.5)	25 (22 to 29)	14 (12 to 16)	28.7 (26.0 to 31.4)	2 (1 to 2)	1 (1 to 1)	28.0 (13.1 to 45.5)
Saint Vincent and the Grenadines	77 (61 to 96)	67 (54 to 85)	46.0 (40.9 to 50.9)	18 (16 to 21)	15 (13 to 17)	47.1 (43.5 to 50.7)	1 (1 to 2)	1 (1 to 1)	46.5 (29.4 to 65.3)
Suriname	358 (286 to 447)	63 (50 to 79)	34.0 (29.4 to 38.6)	84 (73 to 97)	14 (13 to 17)	22.6 (14.4 to 29.0)	5 (3 to 8)	1 (1 to 1)	22.8 (4.2 to 43.5)
Trinidad and Tobago	985 (788 to 1 238)	72 (58 to 90)	20.8 (-4.6 to 40.2)	232 (202 to 268)	15 (13 to 18)	28.0 (12.6 to 40.1)	15 (9 to 22)	1 (1 to 1)	27.4 (6.6 to 50.0)
Virgin Islands	84 (67 to 104)	80 (65 to 98)	41.1 (33.0 to 57.4)	20 (18 to 23)	16 (14 to 19)	33.6 (29.3 to 41.0)	1 (1 to 2)	1 (1 to 2)	33.7 (18.7 to 50.3)
**Central Latin America**	**164 811 (126 716 to 211 666)**	**65 (50 to 84)**	**-2.7 (-12.5 to 4.1)**	**37 303 (32 239 to 44 039)**	**15 (13 to 18)**	**-4.7 (-11.5 to 0.2)**	**2 424 (1 513 to 3 540)**	**1 (1 to 1)**	**-4.6 (-11.8 to 1.3)**
Colombia	28 322 (22 705 to 35 180)	56 (45 to 70)	-24.2 (-37.2 to -14.6)	6 684 (5 774 to 7 821)	13 (11 to 15)	-20.4 (-27.9 to -14.5)	434 (271 to 633)	1 (1 to 1)	-20.6 (-31.8 to -7.4)
Costa Rica	2 689 (2 134 to 3 386)	58 (46 to 73)	26.6 (20.9 to 33.2)	597 (520 to 692)	12 (11 to 15)	24.0 (20.9 to 27.4)	39 (24 to 59)	1 (0 to 1)	24.3 (10.0 to 39.7)
El Salvador	3 530 (2 757 to 4 466)	59 (46 to 74)	-41.7 (-66.6 to -12.2)	1 083 (805 to 1 717)	18 (14 to 30)	-37.6 (-51.8 to -17.8)	68 (42 to 107)	1 (1 to 2)	-37.9 (-53.0 to -14.4)
Guatemala	9 434 (7 455 to 11 770)	58 (45 to 72)	-22.8 (-46.5 to -2.8)	2 129 (1 743 to 2 720)	15 (12 to 20)	-24.3 (-39.5 to -8.1)	138 (86 to 203)	1 (1 to 1)	-24.3 (-41.2 to -1.6)
Honduras	4 849 (3 861 to 6 019)	53 (42 to 66)	7.0 (1.4 to 12.6)	1 024 (869 to 1 219)	13 (11 to 15)	15.8 (8.0 to 29.8)	67 (42 to 97)	1 (1 to 1)	15.3 (-1.3 to 37.6)
Mexico	87 911 (64 642 to 118 936)	71 (52 to 97)	6.5 (0.1 to 12.5)	19 379 (16 541 to 23 111)	16 (14 to 19)	3.5 (0.5 to 6.8)	1 261 (769 to 1 889)	1 (1 to 2)	3.6 (-0.9 to 7.8)
Nicaragua	2 879 (2 269 to 3 651)	46 (36 to 59)	-1.7 (-12.6 to 6.7)	870 (619 to 1 438)	16 (11 to 27)	-30.1 (-43.6 to -10.4)	55 (33 to 89)	1 (1 to 2)	-29.6 (-45.8 to -1.5)
Panama	2 177 (1 738 to 2 739)	56 (45 to 70)	22.1 (15.7 to 28.9)	485 (424 to 561)	12 (11 to 14)	18.4 (12.7 to 22.9)	32 (20 to 47)	1 (0 to 1)	18.1 (5.7 to 31.8)
Venezuela	23 021 (18 415 to 28 435)	75 (60 to 92)	25.0 (16.1 to 35.2)	5 054 (4 371 to 5 850)	16 (14 to 19)	26.0 (19.2 to 34.8)	329 (206 to 486)	1 (1 to 2)	25.7 (10.2 to 45.7)
**Tropical Latin America**	**172 309 (127 267 to 235 272)**	**77 (57 to 105)**	**24.5 (11.5 to 34.1)**	**40 082 (34 217 to 47 980)**	**17 (15 to 21)**	**23.9 (17.4 to 29.1)**	**2 600 (1 585 to 3 904)**	**1 (1 to 2)**	**23.8 (15.9 to 31.2)**
Brazil	167 348 (123 096 to 228 651)	77 (57 to 105)	24.5 (11.4 to 34.4)	39 076 (33 336 to 46 781)	17 (15 to 21)	24.0 (17.3 to 29.3)	2 535 (1 546 to 3 806)	1 (1 to 2)	23.9 (15.7 to 31.5)
Paraguay	4 961 (3 919 to 6 361)	72 (56 to 92)	21.6 (16.7 to 26.2)	1 006 (860 to 1 195)	16 (13 to 18)	21.1 (17.9 to 24.0)	66 (40 to 99)	1 (1 to 2)	20.8 (5.3 to 37.7)
**North Africa and Middle East**	**783 025 (582 997 to 1 151 971)**	**127 (94 to 187)**	**19.9 (0.2 to 54.7)**	**159 838 (123 889 to 221 217)**	**28 (22 to 39)**	**5.0 (-3.1 to 21.7)**	**10 345 (6 319 to 15 711)**	**2 (1 to 3)**	**5.6 (-3.4 to 23.4)**
**North Africa and Middle East**	**783 025 (582 997 to 1 151 971)**	**127 (94 to 187)**	**19.9 (0.2 to 54.7)**	**159 838 (123 889 to 221 217)**	**28 (22 to 39)**	**5.0 (-3.1 to 21.7)**	**10 345 (6 319 to 15 711)**	**2 (1 to 3)**	**5.6 (-3.4 to 23.4)**
Afghanistan	31 927 (25 419 to 40 306)	94 (76 to 119)	-23.4 (-37.4 to -11.9)	10 321 (6 219 to 20 034)	50 (26 to 107)	-30.4 (-36.4 to -19.8)	646 (362 to 1 158)	3 (2 to 6)	-30.3 (-38.3 to -15.8)
Algeria	35 979 (29 269 to 45 071)	88 (72 to 110)	-1.2 (-5.3 to 2.9)	8 053 (7 009 to 9 354)	20 (18 to 24)	-1.2 (-5.4 to 6.0)	523 (327 to 758)	1 (1 to 2)	-1.1 (-13.8 to 13.5)
Bahrain	1 481 (1 204 to 1 853)	99 (81 to 124)	11.3 (5.0 to 17.0)	328 (286 to 380)	21 (19 to 25)	4.5 (0.9 to 8.0)	21 (13 to 32)	1 (1 to 2)	4.9 (-8.6 to 20.7)
Egypt	86 892 (70 964 to 107 197)	88 (72 to 109)	18.9 (12.5 to 27.0)	17 095 (14 754 to 19 908)	19 (17 to 22)	10.0 (6.4 to 14.1)	1 117 (672 to 1 644)	1 (1 to 2)	10.3 (-2.1 to 24.8)
Iran	81 084 (65 990 to 101 119)	98 (80 to 121)	-33.2 (-50.1 to -16.8)	20 907 (17 475 to 26 507)	25 (21 to 32)	-26.7 (-36.8 to -15.9)	1 338 (872 to 1 896)	2 (1 to 2)	-27.1 (-37.9 to -15.9)
Iraq	120 169 (67 097 to 239 652)	266 (148 to 525)	61.5 (3.9 to 157.4)	22 718 (13 391 to 41 289)	59 (35 to 111)	-1.6 (-18.5 to 41.2)	1 461 (768 to 2 740)	4 (2 to 7)	0.1 (-22.2 to 45.1)
Jordan	9 071 (7 357 to 11 393)	82 (67 to 102)	-7.8 (-12.8 to -3.0)	1 741 (1 502 to 2 049)	18 (16 to 21)	-11.1 (-13.8 to -8.3)	114 (70 to 169)	1 (1 to 2)	-11.2 (-21.5 to 0.6)
Kuwait	4 504 (3 640 to 5 719)	103 (84 to 131)	-59.7 (-79.2 to -32.8)	995 (870 to 1 151)	23 (20 to 27)	-42.2 (-63.8 to -21.1)	65 (40 to 95)	2 (1 to 2)	-42.9 (-64.1 to -19.5)
Lebanon	8 928 (7 231 to 11 180)	101 (82 to 127)	-18.7 (-41.6 to 3.3)	2 251 (1 693 to 3 412)	29 (21 to 46)	-26.3 (-40.7 to -6.0)	144 (89 to 220)	2 (1 to 3)	-26.0 (-41.5 to -1.0)
Libya	10 491 (7 642 to 16 021)	147 (107 to 222)	56.3 (21.4 to 136.5)	2 383 (1 687 to 3 771)	35 (25 to 54)	56.2 (19.4 to 127.1)	153 (91 to 244)	2 (1 to 3)	55.0 (15.5 to 132.7)
Morocco	28 893 (23 311 to 36 006)	81 (65 to 101)	6.4 (2.6 to 10.2)	6 439 (5 668 to 7 429)	18 (16 to 21)	2.8 (0.1 to 5.3)	418 (259 to 616)	1 (1 to 2)	2.7 (-9.3 to 17.4)
Palestine	4 685 (3 781 to 5 846)	92 (74 to 113)	-29.7 (-53.1 to -7.4)	1 479 (968 to 2 661)	37 (23 to 69)	-5.8 (-24.0 to 6.0)	93 (55 to 156)	2 (1 to 4)	-7.3 (-27.5 to 10.7)
Oman	5 260 (4 243 to 6 633)	108 (87 to 134)	-0.7 (-6.6 to 5.2)	1 056 (911 to 1 231)	24 (21 to 27)	-5.9 (-9.3 to -2.5)	69 (42 to 104)	2 (1 to 2)	-5.3 (-17.6 to 8.9)
Qatar	3 691 (2 964 to 4 666)	120 (98 to 150)	-4.1 (-8.1 to -0.3)	747 (647 to 872)	26 (23 to 30)	-6.6 (-8.8 to -4.2)	49 (30 to 75)	2 (1 to 3)	-5.7 (-17.4 to 7.1)
Saudi Arabia	48 290 (38 557 to 61 106)	131 (106 to 166)	11.5 (1.0 to 31.8)	9 204 (7 848 to 10 914)	27 (24 to 32)	0.2 (-5.2 to 9.1)	605 (364 to 906)	2 (1 to 3)	1.0 (-11.5 to 15.9)
Sudan	31 586 (24 224 to 41 585)	76 (58 to 99)	-38.8 (-60.4 to -14.4)	6 640 (5 276 to 8 825)	20 (16 to 27)	-16.1 (-37.8 to 0.8)	431 (265 to 643)	1 (1 to 2)	-17.2 (-39.0 to 4.3)
Syria	106 605 (45 416 to 251 316)	588 (248 to 1 389)	724.8 (244.3 to 1 866.0)	15 859 (7 688 to 32 403)	90 (44 to 185)	407.9 (162.2 to 866.4)	1 039 (439 to 2 244)	6 (3 to 13)	415.7 (161.8 to 924.8)
Tunisia	10 387 (8 377 to 13 147)	92 (74 to 116)	-0.2 (-6.9 to 5.7)	2 410 (2 123 to 2 780)	20 (18 to 24)	-3.6 (-7.0 to -0.3)	156 (95 to 231)	1 (1 to 2)	-3.3 (-14.3 to 9.3)
Turkey	76 033 (61 070 to 96 363)	96 (77 to 121)	-3.7 (-9.4 to 4.8)	17 012 (14 862 to 19 694)	20 (18 to 24)	-4.7 (-8.8 to 1.3)	1 100 (689 to 1 625)	1 (1 to 2)	-4.8 (-17.2 to 10.0)
United Arab Emirates	12 860 (10 296 to 16 154)	121 (98 to 151)	-9.3 (-12.5 to -5.9)	2 848 (2 491 to 3 298)	27 (24 to 31)	-10.7 (-12.7 to -8.6)	185 (111 to 286)	2 (1 to 3)	-10.8 (-22.9 to 2.0)
Yemen	63 478 (36 243 to 125 623)	201 (115 to 393)	138.9 (38.9 to 374.8)	9 200 (5 942 to 15 705)	33 (23 to 54)	57.8 (14.2 to 148.9)	606 (317 to 1 131)	2 (1 to 4)	60.8 (12.1 to 166.8)
**South Asia**	**1 443 652 (1 176 395 to 1 791 176)**	**82 (66 to 102)**	**14.8 (9.8 to 19.8)**	**303 065 (264 030 to 353 590)**	**18 (16 to 21)**	**18.1 (15.3 to 20.9)**	**19 715 (12 066 to 28 938)**	**1 (1 to 2)**	**17.9 (13.9 to 22.6)**
**South Asia**	**1 443 652 (1 176 395 to 1 791 176)**	**82 (66 to 102)**	**14.8 (9.8 to 19.8)**	**303 065 (264 030 to 353 590)**	**18 (16 to 21)**	**18.1 (15.3 to 20.9)**	**19 715 (12 066 to 28 938)**	**1 (1 to 2)**	**17.9 (13.9 to 22.6)**
Bangladesh	115 800 (93 862 to 143 607)	73 (60 to 91)	32.6 (25.0 to 39.5)	24 134 (20 774 to 28 226)	16 (14 to 19)	31.8 (27.0 to 37.3)	1 574 (968 to 2 329)	1 (1 to 2)	31.4 (16.5 to 47.9)
Bhutan	803 (650 to 996)	83 (67 to 103)	3.6 (-2.5 to 8.9)	162 (141 to 188)	18 (16 to 21)	2.0 (-1.9 to 6.2)	11 (6 to 15)	1 (1 to 2)	2.0 (-10.9 to 16.6)
India	1 127 439 (912 482 to 1 405 764)	82 (66 to 103)	10.8 (5.3 to 16.3)	239 382 (209 120 to 279 201)	19 (16 to 21)	14.4 (11.6 to 17.2)	15 553 (9 516 to 22 858)	1 (1 to 2)	14.3 (10.1 to 18.7)
Nepal	21 104 (17 237 to 25 975)	71 (57 to 87)	9.5 (5.4 to 13.4)	4 548 (3 932 to 5 277)	17 (15 to 19)	15.3 (10.3 to 23.8)	295 (185 to 428)	1 (1 to 2)	14.8 (-0.2 to 32.5)
Pakistan	178 507 (145 900 to 220 224)	83 (68 to 103)	37.4 (33.0 to 41.8)	34 838 (30 022 to 40 928)	19 (17 to 22)	39.4 (35.0 to 45.3)	2 282 (1 409 to 3 390)	1 (1 to 2)	38.6 (20.2 to 58.6)
**Southeast Asia, East Asia, and Oceania**	**1 496 755 (1 196 902 to 1 864 446)**	**68 (55 to 85)**	**36.9 (26.7 to 47.7)**	**407 812 (365 133 to 459 124)**	**17 (15 to 19)**	**36.9 (31.8 to 41.9)**	**26 426 (16 522 to 38 419)**	**1 (1 to 2)**	**36.5 (30.2 to 42.9)**
**East Asia**	**1 157 965 (916 300 to 1 459 547)**	**78 (62 to 97)**	**48.8 (36.6 to 61.3)**	**324 195 (291 060 to 364 297)**	**19 (17 to 21)**	**45.5 (40.2 to 51.5)**	**20 983 (13 043 to 30 670)**	**1 (1 to 2)**	**45.1 (37.7 to 52.7)**
China	1 104 811 (874 083 to 1 393 248)	78 (62 to 97)	49.8 (37.2 to 62.6)	309 374 (277 663 to 347 850)	19 (17 to 22)	46.1 (40.8 to 52.3)	20 025 (12 458 to 29 263)	1 (1 to 2)	45.8 (38.1 to 53.6)
North Korea	17 172 (13 807 to 21 112)	66 (54 to 81)	51.8 (43.3 to 61.0)	4 549 (4 070 to 5 131)	16 (14 to 18)	53.1 (48.0 to 58.4)	295 (181 to 436)	1 (1 to 2)	52.3 (33.1 to 74.0)
Taiwan (Province of China)	17 327 (13 955 to 21 606)	75 (60 to 93)	5.4 (-4.8 to 15.2)	5 049 (4 544 to 5 659)	18 (16 to 20)	9.3 (3.5 to 14.1)	325 (198 to 476)	1 (1 to 2)	9.0 (-5.3 to 26.6)
**Oceania**	**9 608 (7 838 to 11 569)**	**75 (61 to 91)**	**30.7 (18.4 to 40.1)**	**1 963 (1 703 to 2 270)**	**18 (16 to 21)**	**38.4 (31.8 to 44.0)**	**128 (80 to 185)**	**1 (1 to 2)**	**37.1 (23.4 to 52.5)**
American Samoa	43 (35 to 52)	78 (64 to 95)	25.0 (19.5 to 31.8)	10 (8 to 11)	18 (16 to 21)	27.2 (22.8 to 33.4)	1 (0 to 1)	1 (1 to 2)	26.4 (12.4 to 43.0)
Federated States of Micronesia	77 (63 to 94)	73 (60 to 89)	45.4 (38.5 to 53.2)	17 (14 to 19)	17 (15 to 20)	45.0 (40.5 to 49.9)	1 (1 to 2)	1 (1 to 2)	44.1 (24.3 to 67.1)
Fiji	632 (518 to 769)	69 (56 to 83)	42.2 (34.3 to 51.3)	141 (124 to 161)	16 (14 to 18)	44.2 (39.3 to 49.2)	9 (6 to 14)	1 (1 to 2)	43.0 (25.0 to 63.9)
Guam	148 (121 to 181)	89 (73 to 109)	38.3 (28.9 to 47.0)	36 (32 to 41)	21 (18 to 24)	42.1 (36.4 to 47.5)	2 (1 to 3)	1 (1 to 2)	41.1 (23.3 to 62.1)
Kiribati	70 (58 to 85)	58 (47 to 70)	69.5 (59.2 to 79.0)	14 (13 to 17)	14 (12 to 16)	68.8 (63.1 to 74.7)	1 (1 to 1)	1 (1 to 1)	65.6 (45.2 to 87.5)

Location	Incidence (95% UI)			Prevalence (95% UI)			YLDs (95% UI)		
	2017 counts	2017 age-standardised rates per 100,000	Percentage change in age-standardised rates between 1990 and 2017	2017 counts	2017 age-standardised rates per 100,000	Percentage change in age-standardised rates between 1990 and 2017	2017 counts	2017 age-standardised rates per 100,000	Percentage change in age-standardised rates between 1990 and 2017
Marshall Islands	43<br>(35 to 52)	75<br>(61 to 91)	48.6<br>(41.1 to 56.5)	9<br>(8 to 10)	18<br>(16 to 20)	48.4<br>(43.9 to 53.1)	1<br>(0 to 1)	1<br>(1 to 2)	47.4<br>(27.5 to 68.9)
Northern Mariana Islands	37<br>(30 to 45)	84<br>(69 to 102)	18.0<br>(12.0 to 23.9)	9<br>(8 to 10)	19<br>(17 to 22)	16.7<br>(13.5 to 20.0)	1<br>(0 to 1)	1<br>(1 to 2)	16.9<br>(2.2 to 34.2)
Papua New Guinea	7 074<br>(5 776 to 8 538)	76<br>(62 to 92)	25.9<br>(9.6 to 37.4)	1 423<br>(1 231 to 1 650)	19<br>(16 to 21)	35.4<br>(26.7 to 42.2)	93<br>(57 to 135)	1<br>(1 to 2)	34.1<br>(16.3 to 55.4)
Samoa	145<br>(119 to 175)	74<br>(60 to 90)	36.3<br>(24.1 to 45.8)	31<br>(27 to 36)	18<br>(16 to 20)	46.6<br>(39.4 to 54.6)	2<br>(1 to 3)	1<br>(1 to 2)	45.2<br>(27.5 to 66.8)
Solomon Islands	513<br>(419 to 620)	81<br>(66 to 99)	36.3<br>(31.0 to 41.8)	103<br>(89 to 118)	20<br>(17 to 22)	36.7<br>(33.5 to 40.4)	7<br>(4 to 10)	1<br>(1 to 2)	35.3<br>(18.8 to 54.5)
Tonga	69<br>(57 to 84)	67<br>(55 to 82)	48.0<br>(36.9 to 57.7)	15<br>(13 to 17)	16<br>(14 to 18)	51.8<br>(46.0 to 57.4)	1<br>(1 to 1)	1<br>(1 to 1)	50.1<br>(30.1 to 73.3)
Vanuatu	226<br>(184 to 275)	80<br>(64 to 97)	45.8<br>(38.5 to 53.2)	47<br>(41 to 54)	20<br>(17 to 22)	47.6<br>(43.0 to 52.7)	3<br>(2 to 4)	1<br>(1 to 2)	46.2<br>(27.0 to 68.9)
**Southeast Asia**	**329 183<br>(265 766 to 403 670)**	**49<br>(40 to 60)**	**13.0<br>(-6.2 to 29.3)**	**81 653<br>(71 535 to 94 303)**	**12<br>(11 to 14)**	**16.6<br>(6.9 to 24.7)**	**5 315<br>(3 398 to 7 693)**	**1<br>(1 to 1)**	**16.2<br>(5.3 to 25.8)**
Cambodia	8 575<br>(6 881 to 10 738)	53<br>(42 to 66)	-0.8<br>(-24.6 to 18.7)	2 521<br>(1 961 to 3 701)	17<br>(14 to 26)	-13.6<br>(-30.4 to 7.3)	161<br>(100 to 240)	1<br>(1 to 2)	-13.4<br>(-34.1 to 14.0)
Indonesia	78 421<br>(60 624 to 102 250)	30<br>(24 to 39)	-2.0<br>(-9.6 to 6.5)	21 926<br>(19 352 to 25 469)	9<br>(8 to 10)	0.7<br>(-3.8 to 5.9)	1 432<br>(911 to 2 074)	1<br>(0 to 1)	0.4<br>(-5.6 to 6.8)
Laos	3 425<br>(2 766 to 4 241)	48<br>(39 to 59)	-18.1<br>(-44.9 to 6.1)	761<br>(666 to 877)	12<br>(11 to 14)	-0.6<br>(-20.2 to 12.4)	50<br>(30 to 74)	1<br>(0 to 1)	-1.2<br>(-23.9 to 22.3)
Malaysia	19 322<br>(15 463 to 24 449)	61<br>(49 to 77)	32.3<br>(23.3 to 42.1)	4 546<br>(3 980 to 5 187)	15<br>(13 to 17)	28.9<br>(23.4 to 34.7)	297<br>(183 to 444)	1<br>(1 to 1)	28.7<br>(10.6 to 48.1)
Maldives	243<br>(195 to 301)	49<br>(40 to 60)	24.8<br>(16.8 to 31.8)	54<br>(48 to 63)	12<br>(11 to 13)	21.5<br>(16.7 to 27.2)	4<br>(2 to 5)	1<br>(0 to 1)	21.6<br>(4.9 to 40.0)
Mauritius	612<br>(492 to 754)	49<br>(40 to 60)	54.4<br>(46.2 to 63.7)	168<br>(151 to 189)	12<br>(10 to 13)	52.6<br>(47.8 to 57.6)	11<br>(7 to 16)	1<br>(0 to 1)	51.5<br>(32.1 to 73.0)
Myanmar	53 661<br>(35 743 to 83 624)	98<br>(66 to 151)	161.3<br>(76.9 to 313.5)	9 905<br>(7 584 to 13 604)	19<br>(14 to 25)	81.8<br>(42.2 to 145.7)	647<br>(397 to 1 029)	1<br>(1 to 2)	84.2<br>(38.0 to 151.8)
Philippines	57 754<br>(46 012 to 71 206)	55<br>(44 to 68)	12.6<br>(-5.6 to 30.6)	12 395<br>(10 710 to 14 423)	13<br>(11 to 15)	27.5<br>(14.9 to 37.1)	809<br>(508 to 1 203)	1<br>(1 to 1)	25.8<br>(6.9 to 45.9)
Sri Lanka	10 769<br>(8 726 to 13 146)	50<br>(41 to 61)	-65.9<br>(-84.0 to -33.0)	4 057<br>(3 018 to 6 366)	18<br>(13 to 27)	-22.1<br>(-57.8 to 15.2)	258<br>(156 to 389)	1<br>(1 to 2)	-24.7<br>(-58.7 to 16.7)
Seychelles	58<br>(47 to 72)	56<br>(46 to 69)	46.6<br>(38.6 to 55.0)	15<br>(13 to 16)	14<br>(12 to 15)	44.6<br>(39.7 to 49.3)	1<br>(1 to 1)	1<br>(1 to 1)	43.9<br>(26.0 to 64.3)
Thailand	43 907<br>(34 746 to 54 921)	62<br>(50 to 78)	17.0<br>(10.4 to 24.6)	12 380<br>(11 049 to 13 978)	15<br>(13 to 17)	15.7<br>(11.8 to 19.8)	801<br>(507 to 1 174)	1<br>(1 to 1)	15.7<br>(1.8 to 32.7)
Timor-Leste	551<br>(448 to 668)	43<br>(35 to 52)	-61.0<br>(-81.6 to -23.4)	226<br>(139 to 438)	23<br>(13 to 46)	-15.2<br>(-46.1 to 6.5)	14<br>(8 to 26)	1<br>(1 to 3)	-17.9<br>(-48.0 to 12.4)
Vietnam	51 452<br>(41 604 to 63 669)	52<br>(42 to 65)	42.1<br>(34.8 to 50.0)	12 592<br>(11 136 to 14 333)	13<br>(11 to 14)	38.3<br>(34.4 to 42.9)	822<br>(509 to 1 237)	1<br>(1 to 1)	38.0<br>(20.1 to 55.3)
**Sub-Saharan Africa**	**851 444<br>(699 830 to 1 036 091)**	**85<br>(70 to 104)**	**-26.3<br>(-44.1 to -11.8)**	**168 331<br>(141 460 to 206 341)**	**21<br>(18 to 26)**	**-13.4<br>(-25.9 to -5.9)**	**10 971<br>(6 919 to 15 759)**	**1<br>(1 to 2)**	**-13.9<br>(-26.9 to -5.7)**
**Central sub-Saharan Africa**	**99 549<br>(80 404 to 122 693)**	**84<br>(68 to 104)**	**-4.0<br>(-8.7 to 0.6)**	**20 296<br>(16 624 to 26 184)**	**22<br>(18 to 28)**	**3.1<br>(-4.0 to 16.0)**	**1 317<br>(828 to 1 906)**	**1<br>(1 to 2)**	**2.8<br>(-9.0 to 18.4)**
Angola	21 094<br>(17 144 to 25 961)	79<br>(64 to 99)	-47.3<br>(-67.3 to -26.7)	4 895<br>(3 893 to 6 819)	25<br>(20 to 37)	-29.6<br>(-44.9 to -19.8)	314<br>(199 to 451)	2<br>(1 to 2)	-30.7<br>(-48.6 to -14.9)
Central African Republic	7 943<br>(4 846 to 14 964)	168<br>(104 to 312)	135.7<br>(48.9 to 335.3)	1 277<br>(868 to 2 034)	31<br>(22 to 47)	76.4<br>(30.3 to 169.4)	84<br>(46 to 148)	2<br>(1 to 3)	78.2<br>(27.3 to 180.7)
Congo (Brazzaville)	3 802<br>(3 085 to 4 680)	79<br>(64 to 98)	-7.1<br>(-11.0 to -3.7)	928<br>(752 to 1 252)	23<br>(19 to 31)	10.2<br>(-5.5 to 49.0)	60<br>(37 to 87)	1<br>(1 to 2)	9.0<br>(-11.0 to 51.2)
DR Congo	64 193<br>(51 954 to 78 794)	81<br>(66 to 99)	13.2<br>(4.8 to 28.4)	12 698<br>(10 544 to 15 927)	20<br>(17 to 25)	16.1<br>(4.0 to 42.0)	827<br>(516 to 1 197)	1<br>(1 to 2)	16.4<br>(-3.1 to 48.5)
Equatorial Guinea	1 067<br>(861 to 1 321)	82<br>(66 to 102)	6.2<br>(0.9 to 11.1)	193<br>(166 to 228)	19<br>(17 to 22)	0.4<br>(-2.4 to 3.2)	13<br>(8 to 19)	1<br>(1 to 2)	0.5<br>(-10.7 to 13.9)
Gabon	1 452<br>(1 167 to 1 800)	87<br>(70 to 108)	-14.2<br>(-17.2 to -11.1)	306<br>(267 to 352)	21<br>(18 to 24)	-14.5<br>(-16.3 to -12.7)	20<br>(12 to 29)	1<br>(1 to 2)	-14.4<br>(-23.8 to -2.9)
**Eastern sub-Saharan Africa**	**368 278<br>(297 463 to 456 040)**	**98<br>(79 to 120)**	**-42.2<br>(-61.9 to -20.5)**	**74 997<br>(61 127 to 97 449)**	**26<br>(21 to 34)**	**-22.1<br>(-40.3 to -9.9)**	**4 874<br>(3 097 to 7 018)**	**2<br>(1 to 2)**	**-23.0<br>(-41.4 to -9.5)**
Burundi	9 575<br>(7 802 to 11 793)	92<br>(75 to 113)	-10.4<br>(-15.5 to -2.8)	3 278<br>(2 026 to 6 281)	42<br>(25 to 83)	74.5<br>(5.4 to 242.8)	208<br>(115 to 364)	3<br>(1 to 5)	68.5<br>(2.4 to 236.2)
Comoros	646<br>(519 to 804)	92<br>(74 to 114)	-16.3<br>(-19.6 to -12.8)	132<br>(115 to 153)	21<br>(19 to 24)	-18.1<br>(-20.3 to -16.0)	9<br>(5 to 13)	1<br>(1 to 2)	-17.6<br>(-26.2 to -8.0)
Djibouti	1 059<br>(860 to 1 310)	98<br>(80 to 122)	-27.8<br>(-44.0 to -16.1)	221<br>(192 to 258)	24<br>(21 to 27)	-16.4<br>(-26.9 to -9.6)	14<br>(9 to 21)	2<br>(1 to 2)	-17.1<br>(-31.0 to -3.6)
Eritrea	5 182<br>(4 181 to 6 410)	92<br>(74 to 115)	-94.0<br>(-97.4 to -84.1)	1 974<br>(1 159 to 3 952)	51<br>(27 to 107)	-77.1<br>(-89.4 to -61.9)	123<br>(67 to 227)	3<br>(2 to 6)	-78.5<br>(-90.1 to -61.7)
Ethiopia	89 633<br>(72 465 to 110 542)	90<br>(73 to 111)	-65.6<br>(-81.1 to -41.7)	18 544<br>(14 981 to 24 682)	24<br>(20 to 33)	-43.4<br>(-64.1 to -24.7)	1 205<br>(760 to 1 744)	2<br>(1 to 2)	-44.6<br>(-64.8 to -24.9)
Kenya	48 962<br>(37 752 to 63 083)	107<br>(83 to 139)	13.0<br>(9.4 to 17.9)	9 493<br>(8 058 to 11 307)	25<br>(22 to 29)	11.4<br>(9.1 to 15.0)	621<br>(382 to 913)	2<br>(1 to 2)	11.4<br>(8.2 to 15.7)
Madagascar	23 208<br>(18 758 to 28 693)	93<br>(75 to 115)	-10.6<br>(-14.3 to -6.8)	4 271<br>(3 655 to 5 005)	21<br>(19 to 24)	-12.8<br>(-15.2 to -10.4)	280<br>(170 to 417)	1<br>(1 to 2)	-12.7<br>(-22.6 to -1.1)
Malawi	13 148<br>(10 473 to 16 497)	79<br>(63 to 100)	-2.1<br>(-5.2 to 1.0)	2 418<br>(2 055 to 2 868)	18<br>(16 to 21)	-4.2<br>(-6.4 to -2.3)	159<br>(96 to 234)	1<br>(1 to 2)	-4.1<br>(-14.8 to 8.7)
Mozambique	25 417<br>(20 459 to 31 690)	91<br>(72 to 114)	-22.0<br>(-43.3 to -5.5)	5 286<br>(4 311 to 6 811)	26<br>(21 to 36)	-23.8<br>(-37.7 to -9.8)	342<br>(213 to 495)	2<br>(1 to 2)	-23.7<br>(-39.6 to -4.7)
Rwanda	10 532<br>(8 492 to 13 105)	87<br>(70 to 109)	-54.0<br>(-72.6 to -33.9)	4 009<br>(2 364 to 8 000)	45<br>(25 to 95)	30.1<br>(-28.8 to 157.2)	252<br>(137 to 478)	3<br>(1 to 5)	23.8<br>(-30.5 to 146.6)
Somalia	28 886<br>(19 473 to 49 035)	176<br>(121 to 293)	-3.1<br>(-9.8 to 7.6)	4 738<br>(3 397 to 7 194)	35<br>(27 to 52)	3.7<br>(-3.9 to 13.5)	310<br>(182 to 517)	2<br>(1 to 4)	3.0<br>(-8.3 to 17.9)
South Sudan	20 547<br>(13 379 to 36 318)	214<br>(142 to 373)	45.6<br>(24.1 to 69.9)	3 270<br>(2 278 to 5 060)	41<br>(30 to 61)	41.5<br>(20.0 to 74.7)	214<br>(120 to 369)	3<br>(2 to 4)	41.8<br>(17.0 to 77.7)
Tanzania	45 017<br>(36 126 to 56 211)	87<br>(69 to 109)	3.0<br>(-0.9 to 7.2)	8 296<br>(7 074 to 9 802)	20<br>(17 to 23)	0.0<br>(-2.2 to 2.5)	544<br>(330 to 816)	1<br>(1 to 2)	0.5<br>(-10.4 to 12.7)
Uganda	31 943<br>(25 696 to 40 063)	85<br>(68 to 106)	-5.4<br>(-21.2 to 4.4)	6 404<br>(5 280 to 8 041)	24<br>(19 to 32)	-13.5<br>(-27.0 to -1.4)	416<br>(259 to 597)	2<br>(1 to 2)	-12.7<br>(-29.0 to 6.2)
Zambia	14 295<br>(11 407 to 17 917)	87<br>(69 to 110)	-9.9<br>(-13.6 to -6.4)	2 617<br>(2 238 to 3 093)	20<br>(18 to 23)	-10.4<br>(-12.6 to -8.2)	172<br>(106 to 257)	1<br>(1 to 2)	-10.2<br>(-21.1 to 0.8)
**Southern sub-Saharan Africa**	**67 961<br>(55 412 to 82 604)**	**87<br>(71 to 106)**	**-12.7<br>(-16.3 to -8.9)**	**14 515<br>(12 692 to 16 689)**	**20<br>(18 to 23)**	**-14.2<br>(-16.3 to -12.2)**	**943<br>(599 to 1 364)**	**1<br>(1 to 2)**	**-14.1<br>(-18.2 to -10.1)**
Botswana	1 801<br>(1 466 to 2 200)	79<br>(64 to 96)	-0.4<br>(-4.5 to 3.3)	369<br>(321 to 425)	18<br>(16 to 20)	-0.8<br>(-3.0 to 1.3)	24<br>(15 to 36)	1<br>(1 to 2)	-0.8<br>(-12.1 to 12.0)
Lesotho	1 760<br>(1 443 to 2 127)	89<br>(73 to 109)	20.2<br>(16.1 to 24.2)	357<br>(311 to 414)	21<br>(18 to 23)	20.7<br>(18.0 to 23.2)	23<br>(15 to 34)	1<br>(1 to 2)	20.1<br>(6.4 to 35.1)
Namibia	2 048<br>(1 682 to 2 482)	87<br>(71 to 105)	-5.1<br>(-8.5 to -1.9)	470<br>(392 to 596)	24<br>(20 to 31)	-20.3<br>(-32.7 to -10.3)	30<br>(19 to 44)	1<br>(1 to 2)	-19.6<br>(-35.7 to -2.7)
South Africa	50 482<br>(40 984 to 61 568)	90<br>(74 to 110)	-18.3<br>(-22.5 to -13.9)	11 039<br>(9 673 to 12 680)	21<br>(18 to 24)	-19.5<br>(-22.0 to -17.1)	716<br>(453 to 1 034)	1<br>(1 to 2)	-19.5<br>(-23.9 to -15.0)
Swaziland	1 017<br>(832 to 1 238)	90<br>(73 to 109)	6.3<br>(3.2 to 9.5)	197<br>(170 to 228)	21<br>(18 to 23)	5.0<br>(3.1 to 7.0)	13<br>(8 to 19)	1<br>(1 to 2)	4.8<br>(-7.2 to 18.2)
Zimbabwe	10 853<br>(8 905 to 13 211)	76<br>(62 to 92)	11.0<br>(7.6 to 14.5)	2 083<br>(1 802 to 2 424)	18<br>(16 to 20)	13.0<br>(10.8 to 15.3)	137<br>(84 to 201)	1<br>(1 to 2)	13.1<br>(0.6 to 28.5)
**Western sub-Saharan Africa**	**315 655<br>(259 857 to 382 659)**	**75<br>(62 to 91)**	**-3.3<br>(-8.7 to 0.6)**	**58 522<br>(50 205 to 68 115)**	**17<br>(15 to 20)**	**-1.4<br>(-4.5 to 2.6)**	**3 837<br>(2 360 to 5 549)**	**1<br>(1 to 2)**	**-1.5<br>(-7.5 to 5.7)**
Benin	8 479<br>(6 995 to 10 339)	76<br>(62 to 93)	-3.0<br>(-5.9 to -0.2)	1 575<br>(1 352 to 1 842)	18<br>(16 to 20)	-5.5<br>(-7.1 to -3.8)	104<br>(64 to 153)	1<br>(1 to 2)	-5.2<br>(-16.8 to 7.5)
Burkina Faso	14 345<br>(11 734 to 17 664)	71<br>(58 to 89)	3.1<br>(-0.4 to 7.2)	2 595<br>(2 207 to 3 047)	16<br>(14 to 18)	1.2<br>(-0.9 to 3.4)	171<br>(105 to 251)	1<br>(1 to 2)	1.8<br>(-9.8 to 15.1)
Cameroon	20 587<br>(16 913 to 24 964)	77<br>(63 to 93)	-1.5<br>(-6.7 to 6.3)	3 772<br>(3 235 to 4 409)	17<br>(15 to 20)	-6.7<br>(-9.6 to -2.3)	248<br>(151 to 364)	1<br>(1 to 2)	-6.1<br>(-16.5 to 5.6)
Cape Verde	416<br>(341 to 512)	76<br>(62 to 93)	8.6<br>(5.2 to 12.0)	87<br>(76 to 101)	17<br>(15 to 19)	7.2<br>(5.1 to 9.2)	6<br>(3 to 8)	1<br>(1 to 2)	7.0<br>(-4.2 to 19.3)
Chad	11 239<br>(9 205 to 13 697)	77<br>(63 to 94)	-15.1<br>(-35.8 to 1.6)	2 159<br>(1 796 to 2 683)	20<br>(17 to 26)	-5.5<br>(-18.5 to 4.4)	141<br>(88 to 205)	1<br>(1 to 2)	-6.3<br>(-22.3 to 11.1)
Cote d'Ivoire	17 881<br>(14 702 to 21 968)	74<br>(61 to 91)	-3.5<br>(-6.6 to -0.6)	3 413<br>(2 941 to 3 984)	17<br>(15 to 20)	-3.7<br>(-5.8 to -0.6)	224<br>(138 to 334)	1<br>(1 to 2)	-3.2<br>(-14.6 to 10.3)
The Gambia	1 491<br>(1 228 to 1 833)	71<br>(59 to 88)	2.0<br>(-1.2 to 5.3)	283<br>(243 to 330)	17<br>(15 to 19)	-2.6<br>(-7.6 to 0.4)	19<br>(12 to 28)	1<br>(1 to 2)	-2.2<br>(-14.5 to 11.8)
Ghana	22 251<br>(18 182 to 27 383)	76<br>(62 to 94)	9.9<br>(6.7 to 13.2)	4 348<br>(3 760 to 5 050)	17<br>(15 to 20)	8.1<br>(6.3 to 10.0)	286<br>(174 to 423)	1<br>(1 to 2)	8.7<br>(-3.8 to 22.9)
Guinea	8 291<br>(6 816 to 10 137)	73<br>(60 to 90)	-1.4<br>(-4.6 to 2.0)	1 582<br>(1 364 to 1 842)	17<br>(15 to 20)	-1.2<br>(-3.8 to 2.3)	104<br>(63 to 151)	1<br>(1 to 2)	-1.1<br>(-12.5 to 12.3)
Guinea-Bissau	1 231<br>(1 011 to 1 508)	68<br>(56 to 84)	-12.9<br>(-15.9 to -10.1)	234<br>(201 to 274)	17<br>(15 to 19)	-11.1<br>(-14.4 to -4.4)	15<br>(9 to 23)	1<br>(1 to 2)	-11.2<br>(-21.7 to 2.4)
Liberia	2 934<br>(2 395 to 3 615)	64<br>(52 to 78)	-87.2<br>(-94.6 to -68.3)	749<br>(561 to 1 151)	21<br>(16 to 33)	-66.7<br>(-85.4 to -38.2)	48<br>(29 to 73)	1<br>(1 to 2)	-68.5<br>(-85.5 to -39.5)
Mali	16 204<br>(13 123 to 20 192)	82<br>(66 to 102)	-2.6<br>(-5.9 to 1.0)	3 026<br>(2 490 to 3 827)	20<br>(16 to 25)	4.6<br>(-4.9 to 26.5)	198<br>(125 to 292)	1<br>(1 to 2)	4.3<br>(-11.9 to 29.7)
Mauritania	2 689<br>(2 193 to 3 320)	70<br>(57 to 87)	-12.0<br>(-16.4 to -8.4)	510<br>(438 to 596)	16<br>(14 to 18)	-12.9<br>(-15.0 to -10.9)	33<br>(20 to 49)	1<br>(1 to 2)	-12.6<br>(-22.3 to -1.6)
Niger	14 866<br>(12 186 to 18 065)	73<br>(60 to 89)	-8.8<br>(-13.0 to -5.0)	2 572<br>(2 180 to 3 037)	17<br>(15 to 19)	-9.1<br>(-11.5 to -6.8)	169<br>(103 to 250)	1<br>(1 to 2)	-9.4<br>(-19.7 to 3.1)
Nigeria	151 936<br>(124 561 to 185 566)	76<br>(62 to 92)	7.7<br>(1.7 to 17.3)	27 450<br>(23 430 to 32 080)	17<br>(15 to 19)	3.3<br>(-0.3 to 9.3)	1 801<br>(1 096 to 2 617)	1<br>(1 to 2)	3.2<br>(-8.4 to 17.0)
Sao Tome and Principe	152<br>(121 to 190)	77<br>(61 to 97)	3.7<br>(-0.5 to 8.0)	29<br>(25 to 35)	18<br>(15 to 20)	1.7<br>(-0.7 to 4.1)	2<br>(1 to 3)	1<br>(1 to 2)	1.6<br>(-9.8 to 14.8)
Senegal	10 045<br>(8 239 to 12 365)	71<br>(58 to 87)	-9.3<br>(-15.3 to -5.2)	1 931<br>(1 664 to 2 251)	16<br>(15 to 19)	-8.0<br>(-10.9 to -5.4)	126<br>(77 to 185)	1<br>(1 to 2)	-8.0<br>(-19.4 to 4.6)
Sierra Leone	5 496<br>(4 505 to 6 673)	72<br>(59 to 88)	0.6<br>(-4.2 to 8.6)	1 227<br>(984 to 1 665)	20<br>(16 to 29)	20.3<br>(0.7 to 66.9)	79<br>(49 to 117)	1<br>(1 to 2)	19.3<br>(-4.6 to 66.1)
Togo	5 118<br>(4 209 to 6 281)	69<br>(57 to 85)	-0.7<br>(-4.0 to 2.6)	979<br>(845 to 1 142)	16<br>(14 to 18)	-3.0<br>(-4.9 to -0.9)	64<br>(39 to 95)	1<br>(1 to 2)	-2.5<br>(-14.5 to 12.0)
